# Genome-wide identification and characterization of long non-coding RNAs involved in flag leaf senescence of rice

**DOI:** 10.1007/s11103-021-01121-3

**Published:** 2021-02-11

**Authors:** Xiaoping Huang, Hongyu Zhang, Qiang Wang, Rong Guo, Lingxia Wei, Haiyan Song, Weigang Kuang, Jianglin Liao, Yingjin Huang, Zhaohai Wang

**Affiliations:** 1 Key Laboratory of Crop Physiology, Ecology and Genetic Breeding (Jiangxi Agricultural University), Ministry of Education of the P.R. China, Nanchang, 330045 Jiangxi Province China; 2 Key Laboratory of Agriculture Responding to Climate Change (Jiangxi Agricultural University), Nanchang City, 330045 Jiangxi Province China; 3 Southern Regional Collaborative Innovation Center for Grain and Oil Crops in China, Changsha, 410128 Hunan Province China

**Keywords:** Competing endogenous RNA (ceRNA), Long non-coding RNAs (lncRNAs), Leaf senescence, Rice, Weighted gene co-expression network analysis (WGCNA)

## Abstract

**Key message:**

This study showed the systematic identification of long non-coding RNAs (lncRNAs) involving in flag leaf senescence of rice, providing the possible lncRNA-mRNA regulatory relationships and lncRNA-miRNA-mRNA ceRNA networks during leaf senescence.

**Abstract:**

LncRNAs have been reported to play crucial roles in diverse biological processes. However, no systematic identification of lncRNAs associated with leaf senescence in plants has been studied. In this study, a genome-wide high throughput sequencing analysis was performed using rice flag leaves developing from normal to senescence. A total of 3953 lncRNAs and 38757 mRNAs were identified, of which 343 lncRNAs and 9412 mRNAs were differentially expressed. Through weighted gene co-expression network analysis (WGCNA), 22 continuously down-expressed lncRNAs targeting 812 co-expressed mRNAs and 48 continuously up-expressed lncRNAs targeting 1209 co-expressed mRNAs were considered to be significantly associated with flag leaf senescence. Gene Ontology results suggested that the senescence-associated lncRNAs targeted mRNAs involving in many biological processes, including transcription, hormone response, oxidation–reduction process and substance metabolism. Additionally, 43 senescence-associated lncRNAs were predicted to target 111 co-expressed transcription factors. Interestingly, 8 down-expressed lncRNAs and 29 up-expressed lncRNAs were found to separately target 12 and 20 well-studied senescence-associated genes (SAGs). Furthermore, analysis on the competing endogenous RNA (CeRNA) network revealed that 6 down-expressed lncRNAs possibly regulated 51 co-expressed mRNAs through 15 miRNAs, and 14 up-expressed lncRNAs possibly regulated 117 co-expressed mRNAs through 21 miRNAs. Importantly, by expression validation, a conserved miR164-NAC regulatory pathway was found to be possibly involved in leaf senescence, where lncRNA MSTRG.62092.1 may serve as a ceRNA binding with miR164a and miR164e to regulate three transcription factors. And two key lncRNAs MSTRG.31014.21 and MSTRG.31014.36 also could regulate the abscisic-acid biosynthetic gene BGIOSGA025169 (*OsNCED4*) and BGIOSGA016313 (NAC family) through osa-miR5809. The possible regulation networks of lncRNAs involving in leaf senescence were discussed, and several candidate lncRNAs were recommended for prior transgenic analysis. These findings will extend the understanding on the regulatory roles of lncRNAs in leaf senescence, and lay a foundation for functional research on candidate lncRNAs.

**Supplementary Information:**

The online version contains supplementary material available at 10.1007/s11103-021-01121-3.

## Introduction

Non-coding RNAs are the main products of the eukaryotic transcriptome and possess key regulatory functions (Fabbri and Calin 2010). Long non-coding RNAs (lncRNAs) are a class of endogenous non-coding RNAs longer than 200 nucleotides which lack apparent protein-coding capacity (Zhu and Wang 2012). Based on the location in the genome, lncRNAs can be classified into five groups, including sense, antisense, intronic, bidirectional and long intergenic non-coding RNAs (lincRNAs) (Mattick and Rinn 2015). Compared with protein-coding genes, most lncRNAs are usually expressed at low levels and lack strong sequence conservation between species (Cabili et al. 2011; Necsulea et al. 2014). Increasing evidence has shown that lncRNAs play vital functions on growth, development, disease occurrence and epigenetic regulation in mammals (Sleutels et al. 2002; Rinn et al. 2007; Zhao et al. 2008).

With the rapid advances in high-throughput sequencing, numerous lncRNAs have been identified in plant species. For example, a total of 3857 lncRNAs have been identified during fruit ripening in melon, of which 1601 were differentially expressed between developmental stages and 142 are highly expressed (Tian et al. 2019). A comprehensive view of 833 high-confidence lncRNAs including 652 intergenic and 181 antisense lncRNAs in cassava have been identified under drought stress condition, of which 124 are drought-responsive (Ding et al. 2019). Additionally, 2572 lncRNAs related to flower development have been identified in *Prunus mume*, of which two lncRNAs XR_514690.2 and TCONS_00032517 might contribute to the formation of multiple pistils (Wu et al. 2019). In non-heading Chinese cabbage, a total of 4594 putative lncRNAs have been identified with a comprehensive landscape of dynamic lncRNA expression networks under heat stress (Wang et al. 2019a). Moreover, recent studies have also reported the functional studies of lncRNAs in plants. For example, overexpression of lncRNA T5120 in *Arabidopsis* promotes the response to nitrate, enhances nitrate assimilation, and improves biomass and root development (Liu et al. 2019). A total of 567 disease-responsive lncRNAs in rice have been systematically identified, among which, overexpression of lncRNA ALEX1 could activate jasmonate pathway and enhance resistance to bacterial blight (Yu et al. 2020). Taken together, these results all show the essential functions of lncRNAs in various biological processes, but not decipher in leaf senescence.

Emerging evidence has shown that lncRNAs could regulate the expression of protein-coding genes via numerous complex mechanisms to execute their functions (Liu et al. 2015a). For example, a novel antisense long noncoding RNA, *TWISTED LEAF*, maintains leaf blade flattening by regulating its associated sense gene *R2R3-MYB* in rice (Liu et al. 2018). A tomato lncRNA 16397 could regulate *SlGRX* gene expression to decrease ROS accumulation and weaken the injury of cell membrane, leading to increased resistance to *Phytophthora infestans* (Cui et al. 2017). In addition, a rice long non-coding RNA *Ef-cd* transcribed from the antisense strand of the flowering activator *OsSOC1* locus can positively regulate the expression of *OsSOC1*, and plays roles in balancing grain yield with maturity duration (Fang et al. 2019). Overexpressing lncRNA LAIR transcribed from the antisense strand of neighbouring gene *LRK* (leucine-rich repeat receptor kinase) could increase grain yield and regulate the expression of *LRK* gene cluster in rice (Wang et al. 2018a). A rice *cis*-natural antisense long non-coding RNA *cis*-NAT_*PHO1;2*_ acts as a translational enhancer for its cognate mRNA and contributes to phosphate homeostasis and plant fitness (Jabnoune et al. 2013). Moreover, it has been reported that lncRNAs could act as a class of competing endogenous RNAs (ceRNAs) binding with microRNAs (miRNAs) to condense its repression on target genes (Zhu and Wang 2012). One example of ceRNA was found in *Arabidopsis*, and the study showed that lncRNA IPS1 could influence the expression level of *PHO2* by binding to miR399 (Franco-Zorrilla et al. 2007). In addition, 89 lncRNA-originated endogenous target mimics for 46 miRNAs in tomato have been identified, of which lncRNA *23468* functions as a competing endogenous RNA to modulate *NBS-LRR* genes by decoying miR482b in the tomato *Phytophthora infestans* interaction (Jiang et al. 2019). A long non-coding RNA osa-eTM160 could attenuate the repression of osa-miR160 on osa-ARF18 mRNA during early anther developmental stages (Wang et al. 2017). Besides, ceRNA analysis also have been clearly illustrated in other species, such as honey bee (Chen et al. 2017), cucumber (He et al. 2019) and pepper (Zuo et al. 2019). However, ceRNA network has not been analyzed to study the functions of lncRNAs involved in leaf senescence.

Leaf senescence is a genetically programmed cell death process that constitutes the final stage of leaf development (Leng et al. 2017). Leaf senescence may limit yield in crop plants (Lim et al. 2007). Rice (*Oryza sativa* L.), a monocotyledonous model organism, is one of the major food crops. Thus, deciphering leaf senescence in rice is of great importance for understanding its molecular regulatory mechanism, and provides means to control leaf senescence for genetic improvement of yield. In the past decades, enough efforts to reveal the molecular mechanisms underlying leaf senescence have been mainly made by transcriptomic (mRNA), proteomic and metabolomic researches (Kim et al. 2016). Certainly, the identification of leaf senescence-associated genes (SAGs) and their functional studies were also performed, such as *ONAC011* (El Mannai et al. 2017), *SPL29* (Wang et al. 2015) and *OsCPK12* (Wang et al. 2019b). However, no systematic identification of lncRNAs involved in leaf senescence in rice has been reported.

In this study, a genome-wide high throughput sequencing approach was applied to investigate the genome-wide transcriptome changes of rice flag leaves during the developing process to senescence. Subsequently, senescence-related lncRNAs were systematically identified and characterized. Integrating the analysis on target gene functions and ceRNA networks, the putative regulatory function of these lncRNAs to mRNAs were predicted and analyzed. These results provided new insights into the regulatory mechanism of lncRNAs in rice during flag leaf senescence, and laid a foundation for functional research on the candidate lncRNAs.

## Materials and methods

### Plant material and sample collection

The standard laboratory rice cultivar 93-11 (*Oryza sativa* L.), an excellent maintainer line of indica rice in China, was used as the experimental material in this study. The 93-11 seeds were maintained in our laboratory by strict selfing.

**Fig. 1:**
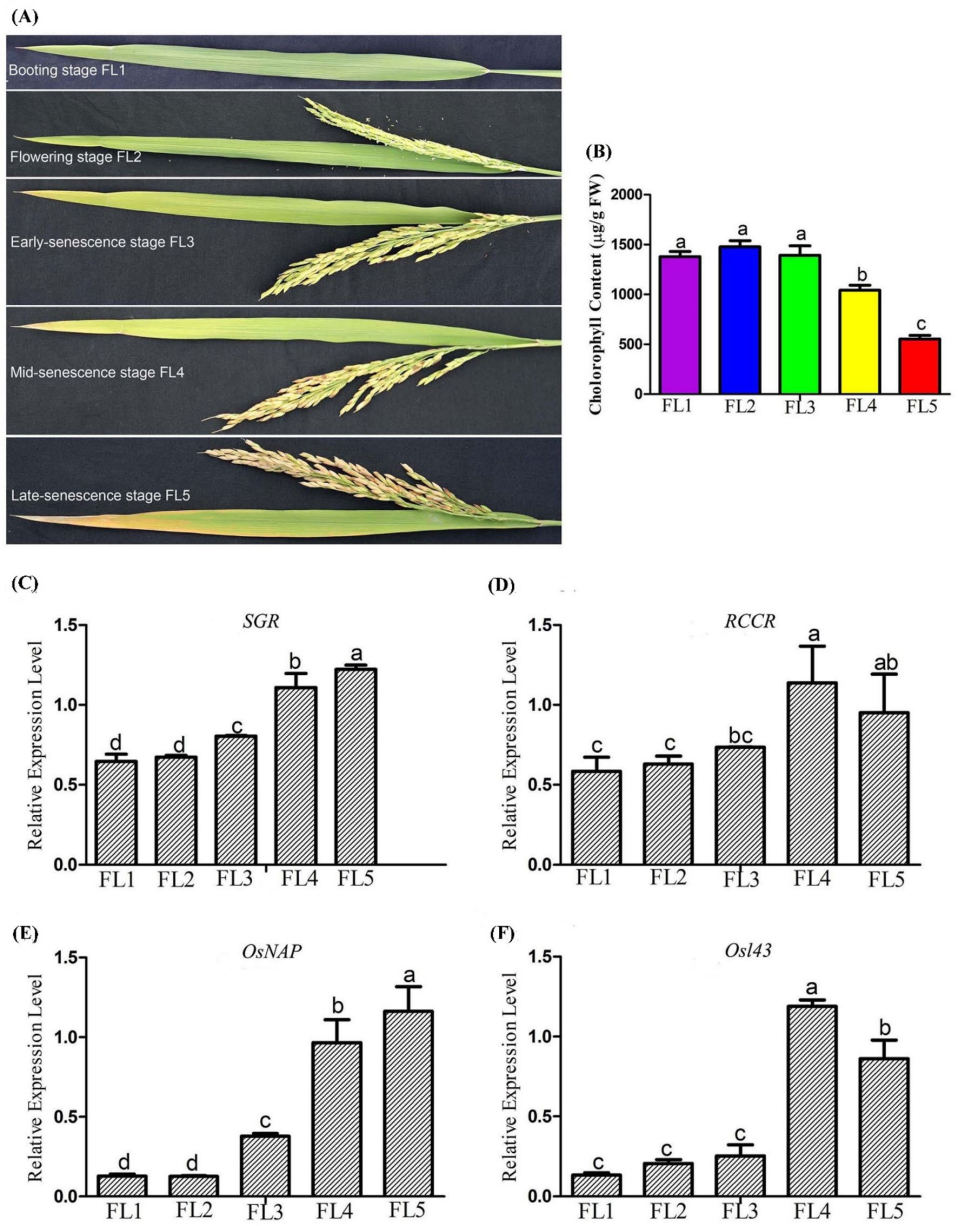
The senescence feature of rice flag leaves sampled at five developmental stages. **a** The sampled flag leaves at booting stage (FL1), flowering stage (FL2), early-senescence stage (FL3), mid-senescence stage (FL4) and late-senescence stage (FL5), respectively. **b** The measured chlorophyll content of rice flag leaf. **c**, **d** The expression trends of two chlorophyll degradation-associated genes (*SGR*, *LOC_Os09g36200*; *RCCR1*, *LOC_Os10g25030*). **e**, **f** The expression trends of two senescence-associated genes (*OsNAP*, *LOC_Os03g21060*; *Osl43*, *LOC_Os01g24710*). The data are expressed as the mean ± SD of three biological replicates. Different letters indicate values are statistically different based on one-way ANOVA analysis

The 93-11 plants were cultivated in paddy field by normal management way. The flag leaves at booting stage (FL1, 9 days before flowering), flowering stage (FL2, 3 days after flowering), early-senescence stage (FL3, 9 days after flowering), mid-senescence stage (FL4, 19 days after flowering) and late-senescence stage (FL5, 29 days after flowering) were collected, respectively (Fig. 1a). Three biological replicates were used for samples in each stage, with each sample pooled with three flag leaves from three independent plants, flash-frozen in liquid nitrogen, and stored at − 80 °C for subsequent analysis.

### Detection of chlorophyll content and senescence marker genes

Chlorophyll was extracted from rice flag leaves with ice-cold 80% acetone, and the chlorophyll content per gram of leaf fresh weight (FW) was determined as previously described (Lichtenthaler 1987). Furthermore, the expression levels of senescence marker genes *SGR* (*LOC_Os09g36200*), *RCCR1* (*LOC_Os10g25030*), *OsNAP* (*LOC_Os03g21060*) and *Osl43* (*LOC_Os01g24710*) (Table S1) were performed by quantitative real-time polymerase chain reaction (qRT-PCR).

### RNA extraction, library construction and sequencing

Total RNAs from samples were extracted with TRIzol reagent (Invitrogen, USA). Subsequently, the quality and integrity of total RNAs were detected by using Nanodrop 2000 and Agilent Bioanalyzer 2100 System (Agilent Technologies, USA).

After RNA extraction, a total of 1.5 μg RNA per sample was firstly used to remove ribosomal RNA using a Ribo-Zero rRNA Removal Kit (Epicentre, Madison, USA). Then, the sequencing libraries were constructed using NEBNext® Ultra™ Directional RNA Library Prep Kit for Illumina® (NEB, USA) according to the manufacturer’s recommendations. Finally, the libraries were sequenced on an Illumina HiSeq 2500 platform, and 150 bp paired-end reads were generated.

### Identification of lncRNAs and mRNAs

After RNA-Seq, clean reads were obtained by trimming adaptor sequences and removing low quality reads from raw data. Then, the clean reads were mapped to the rice reference genome based on MSU-v7.0 (http://rice.plantbiology.msu.edu/) using HISAT2 software (Kim et al. 2015). Next, the mapped reads were assembled using the StringTie software (Pertea et al. 2016). Then, the assembled transcripts were annotated using the gffcompare program. Finally, the protein-coding transcripts were identified and the remaining unknown transcripts were used to screen for putative lncRNAs.

For lncRNAs identification, the screening criteria were as follows: (1) the transcripts with a single exon and less than 200 bp long were removed; (2) the transcripts that were identified as known mRNAs and other small RNAs were removed; (3) the transcripts with protein-coding ability were removed based on the evaluation of coding potential calculator (CPC, CPC score > 0) (Kong et al., 2007), coding-non-coding index (CNCI, CNCI score > 0) (Sun et al., 2013), and coding potential assessing tool (CPAT) (Wang et al. 2013); (4) the transcripts with known protein domains were also excluded according to protein family database (Pfam) (Finn et al. 2013). Finally, the remaining transcripts were considered as reliable lncRNAs. The different types of lncRNAs, including long intergenic noncoding RNAs (lincRNAs), antisense lncRNAs, intronic lncRNAs and sense lncRNAs were classified based on their location on the genome. Furthermore, the sequences of plant lncRNAs were downloaded from the Green Non-Coding Database (GreeNC) for sequence conservation analysis (Wang et al. 2019a).

### Identification of differentially expressed (DE) lncRNAs and DE mRNAs

The fragments per kilobase of transcript per million mapped reads value calculated by StringTie software was used to quantify the expression levels of both lncRNAs and mRNAs in each sample. A pairwise differential expression analysis between any two stages (FL1 vs FL2; FL1 vs FL3; FL1 vs FL4; FL1 vs FL5; FL2 vs FL3; FL2 vs FL4; FL2 vs FL5; FL3 vs FL4; FL3 vs FL5; FL4 vs FL5) was performed using the DESeq R package (1.10.1). LncRNAs and protein-coding mRNAs with a false discovery rate < 0.05 and absolute value of log2 (Fold change) > 1 found by DE Seq were considered to be differentially expressed. Furthermore, hierarchical clustering analysis on DE lncRNAs and DE mRNAs were carried out using heatmap R package.

### Target gene prediction of lncRNAs

Potential target genes of the lncRNAs were predicted based on their regulatory patterns, which were classified into *cis*- and *trans*-acting groups (Stadler 2014). These protein coding genes transcribed within a 100 kb upstream or downstream of lncRNAs were searched as potential *cis* target genes by using Perl script (Tian et al. 2019). Furthermore, the *trans*-acting target genes were determined by calculating the expression correlation between the expression level of lncRNAs and mRNAs. And the correlation in expression was evaluated using Pearson’s correlation coefficient (|r|> 0.9 and *p* < 0.01) (Tian et al. 2019).

### Co-expression network analysis of DE lncRNAs and DE mRNAs

To further explore the functions of lncRNAs related to leaf senescence, a weighted gene co-expression network analysis (WGCNA) was conducted using the R package WGCNA (Langfelder and Horvath 2008). An unsigned co-expression relationship was built based on the adjacency matrix between DE lncRNAs and DE mRNAs. The one-step network construction and module detection were adopted using the “dynamic hybrid tree cut algorithm” with the power value of 5, minimum module size of 30 and merge cut height of 0.2826. The other parameters were defined as default values. Highly similar modules were subsequently identified by clustering and then merged into new modules on the basis of eigengenes. The correlation of each module was also analyzed and visualized by a heatmap. Finally, the co-expression network was visualized by Cytoscape software.

### Function classification for the target mRNAs of interested lncRNAs

Gene Ontology (GO) enrichment analysis of the target mRNAs which co-expressed with interested lncRNAs were implemented using Blast2GO (Conesa et al. 2005), and GO terms with *p*-value < 0.05 were considered significantly enriched with differential expressed mRNAs. We used R package to test the statistical enrichment of the target mRNAs which co-expressed with interested lncRNAs in Kyoto Encyclopedia of Genes and Genomes (KEGG) pathways. The *p*-value < 0.05 was required for differences to be considered statistically significant. The KEGG pathways were enlisted in order according to the corrected p value, and pathways with a corrected *p*-value < 0.05 were considered to be most enriched.

### Construction of lncRNAs–miRNAs–mRNAs network

To explore the coordinated functions of DE lncRNAs, the ceRNA networks were constructed. Firstly, known mature miRNA sequences for rice were downloaded from miRBase (release 22, October 2018). Subsequently, the identified DE lncRNAs and DE mRNAs in ‘ME black’ and ‘ME blue’ module were used as target prediction library. Then, psRNATarget was used with default parameters to identify miRNA target binding sites (Dai et al. 2018). And regulatory data of miRNAs-DE lncRNAs and miRNAs-DE mRNAs were obtained. In addition, the target mRNAs were compared with the DE mRNAs in specific modules to obtain the crossover mRNAs. Finally, based on interaction relationship among DE lncRNAs–miRNAs–DE crossover mRNAs, ceRNA networks were constructed and visualized by Cytoscape software.

### Quantitative real-time polymerase chain reaction (qRT-PCR)

To validate the relative expression level of lncRNAs and mRNAs, qRT-PCR analysis was performed. Firstly, the total RNAs of flag leaf samples at five developmental stages in rice were respectively extracted using TRIzol reagent (Invitrogen, USA). Next, random hexamers were used for cDNA synthesis of lncRNAs and mRNAs. Then, qRT-PCR was performed using RNA-specific primers with the SYBR Green PCR kit (TaKaRa, China) according to the manufacturer’s instructions (Table S1). Three genes, *UBC* (*LOC_Os02g42314*), *ARF* (*LOC_Os05g41060*) and *Profilin-2* (*LOC_Os06g*05880) (Wang et al. 2016), were used as internal reference genes to normalize the qRT-PCR data for mRNAs and lncRNAs. The relative RNA expression levels were calculated by using the 2^−ΔΔCT^ method (Ren et al. 2018). The reaction was carried out using three biological replicates with three technical replicates.

## Results

### Analysis of senescence feature during flag leaf development

Chlorophyll degradation is a hallmark feature of leaf senescence. To effectively examine the leaf senescence status, we initially measured the chlorophyll content of rice flag leaves at five developmental stages, including booting stage (FL1, 9 days before flowering), flowering stage (FL2, 3 days after flowering), early-senescence stage (FL3, 9 days after flowering), mid-senescence stage (FL4, 19 days after flowering), and late-senescence stage (FL5, 29 days after flowering) (Fig. 1a). Accompanying with the leaf development from early-senescence stage (FL3) to late senescence stage (FL5), the chlorophyll content significantly decreased from 1392 to 553 μg/g FW (Fig. 1b). To confirm this tendency, the mRNA expression levels of two chlorophyll degradation associated genes *SGR* and *RCCR1* were measured by qRT-PCR and found to increase during the senescence stages (Fig. 1c, d). Meanwhile, the senescence associated genes *OsNAP* and *Osl43*, were also higher expressed in the flag leaves of the senescence stage (Fig. 1e, f). These results suggested that there existed apparent leaf developmental differences among analyzed samples before and after senescence, implying that samples at these time points were suitable for subsequent study.

### High throughput sequencing

To systematically identify lncRNAs and mRNAs related to leaf senescence, a genome-wide high throughput sequencing was performed for rice flag leaf at five developmental stages with the senescence feature identification described above. Sequencing was done on the Illumina HiSeq 2500 platform and 150 bp paired-end reads were generated. After filtering the adapters and low-quality reads, a total of 288.77 gigabases clean reads were obtained from 15 sequencing libraries (5 samples × 3 replicates) (Table 1). And clean reads in each library mapped to the *Oryza sativa* reference genome ranged from 93.75 to 96.36% (Table 1). Furthermore, the Q30 of the clean reads was greater than 94.10% and GC content of the sequencing outputs were 45.67–46.95% (Table 1).

**Table 1:** Statistical analysis of the RNA-Seq reads for five flag leaf samples with three biological replicates

#Sample	Clean Reads	Mapped reads	Q20 (%)	Q30 (%)	GC (%)
FL1-1	123393578	118749129(96.24%)	98.11	94.29	45.67
FL1-2	114930654	110519849(96.16%)	98.22	94.6	45.85
FL1-3	124927304	120382736(96.36%)	98.22	94.55	46.22
FL2-1	131360464	125803133(95.77%)	98.22	94.54	46
FL2-2	130654926	124979781(95.66%)	98.04	94.1	46.01
FL2-3	122224704	116638729(95.43%)	98.21	94.53	46.77
FL3-1	126829020	119701640(94.38%)	98.22	94.53	46.41
FL3-2	159242022	150,956,156(94.80%)	98.22	94.5	46.35
FL3-3	128791336	121,940,027(94.68%)	98.21	94.5	46.52
FL4-1	122445208	115788125(94.56%)	98.41	94.96	46.67
FL4-2	143316646	135235845(94.36%)	98.27	94.64	46.89
FL4-3	123146180	116235245(94.39%)	98.17	94.42	46.37
FL5-1	128511780	120998893(94.15%)	98.1	94.23	46.93
FL5-2	125910476	118612499(94.20%)	98.29	94.65	46.56
FL5-3	136795808	128246831(93.75%)	98.15	94.39	46.95

### Identification of lncRNAs and mRNAs involved in flag leaf senescence of rice

**Fig. 2:**
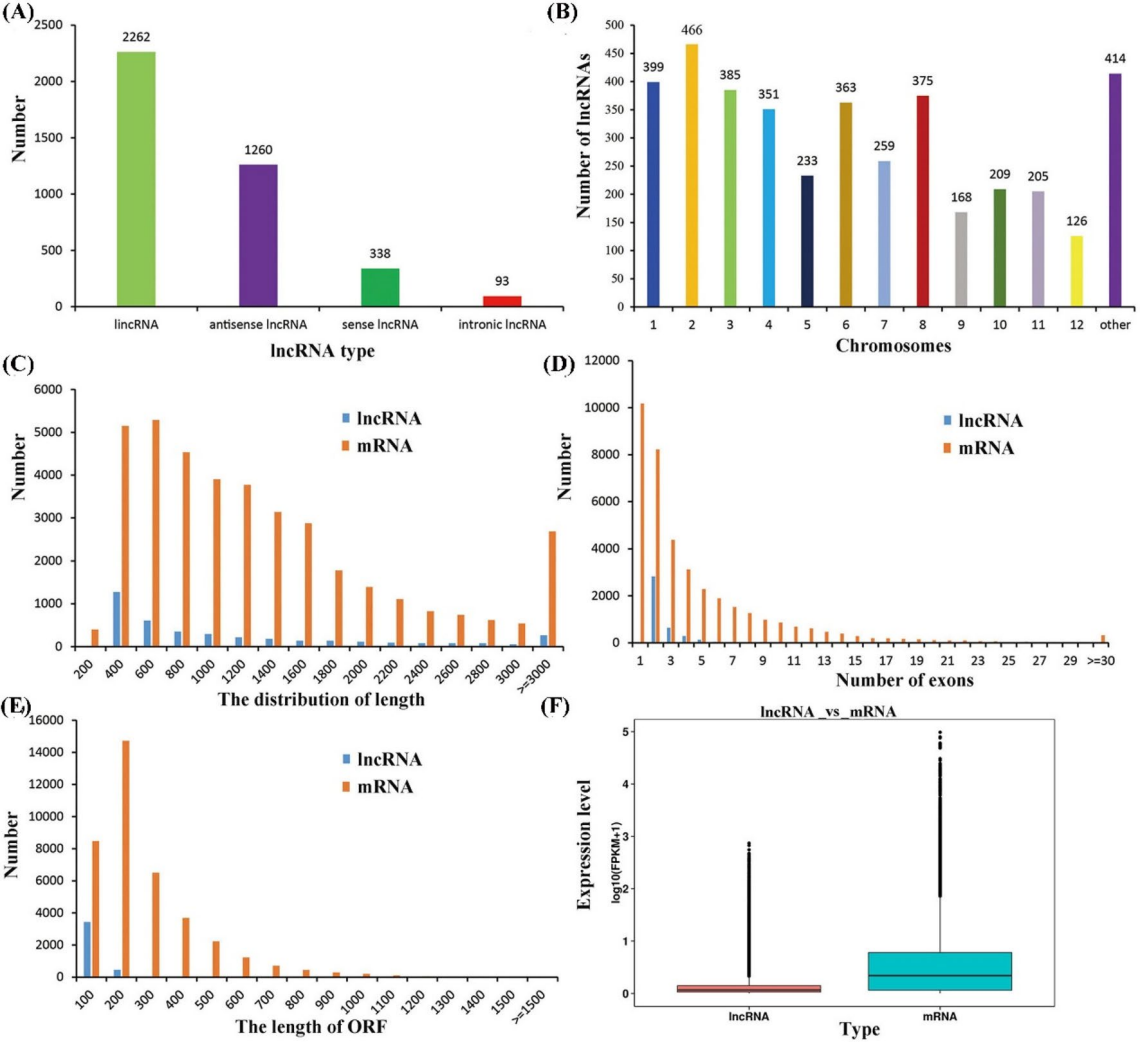
Information of the identified lncRNAs and mRNAs. **a** The types of the identified lncRNAs. **b** The distribution of the identified lncRNAs on chromosome. **c** The length distribution of the identified lncRNAs and mRNAs. **d** The exon distribution of the identified lncRNAs and mRNAs. **e** The length distribution of open reading frame for the identified lncRNAs and mRNAs. **f** The comparison of expression levels between lncRNAs and mRNAs

After clean reads mapping to the *Oryza sativa* reference genome, the mapped transcripts were assembled and annotated using StringTie software (v1.3.1). And a total of 38757 mRNAs were identified (Table S2). The remaining transcripts were filtered. Firstly, the transcripts with length less than 200 bp were removed. Secondly, transcripts with coding potential, which were evaluated by CNCI, CPC, CPAT and Pfam, were removed. At last, a total of 3953 lncRNAs from rice flag leaf at five developmental stages were identified, including long intergenic non-coding RNAs (lincRNAs) (2262, 57.2%), antisense lncRNAs (1260, 31.9%), sense lncRNAs (338, 8.55%) and intronic lncRNAs (93, 2.35%) according to the locations of lncRNAs in the genome (Fig. 2a, Table S3). These identified lncRNAs were distributed on all chromosomes with the largest number on the chromosome 2 (Fig. 2b, Table S3). Most importantly, the conservation analysis of lncRNAs was performed with the criteria E value < 1e-5. The results showed that 1, 4, 11, 11 and 57 lncRNAs have sequence conservation with *Brachypodium distachyon*, *Zea mays*, *Setaria italica*, *Triticum aestivum* and *Sorghum bicolor*, respectively. And 3, 18, 18, 23 and 63 lncRNAs showed sequence conservation with *Medicago truncatula*, *Arabidopsis thaliana*, *Populus trichocarpa*, *Cucumis sativus* and *Manihot esculenta*, respectively (Table S4). Accordingly, the result further indicated the low conservation of lncRNAs among different organisms.

The basic genomic features of these lncRNAs were additionally characterized. Compared with the mRNAs, the lncRNAs were mainly 400–1600 nt in length, and the number of lncRNAs decrease with the increasing transcript length (< 3000 nt) (Fig. 2c). The number of the corresponding exons of lncRNAs was much less than that of mRNAs and mainly below 5 (Fig. 2d), while that of mRNAs ranging from 1 to 30 (Fig. 2d). The length of open reading frames of lncRNAs was mainly below 100 nt (Fig. 2e), while that of mRNAs was mainly below 900 nt (Fig. 2e). In addition, the expression level of mRNAs was higher than those of lncRNAs (Fig. 2f). These results thus revealed divergent features of lncRNAs compared with those of mRNAs.

### Analysis of differentially expressed (DE) lncRNAs and DE mRNAs

**Fig. 3:**
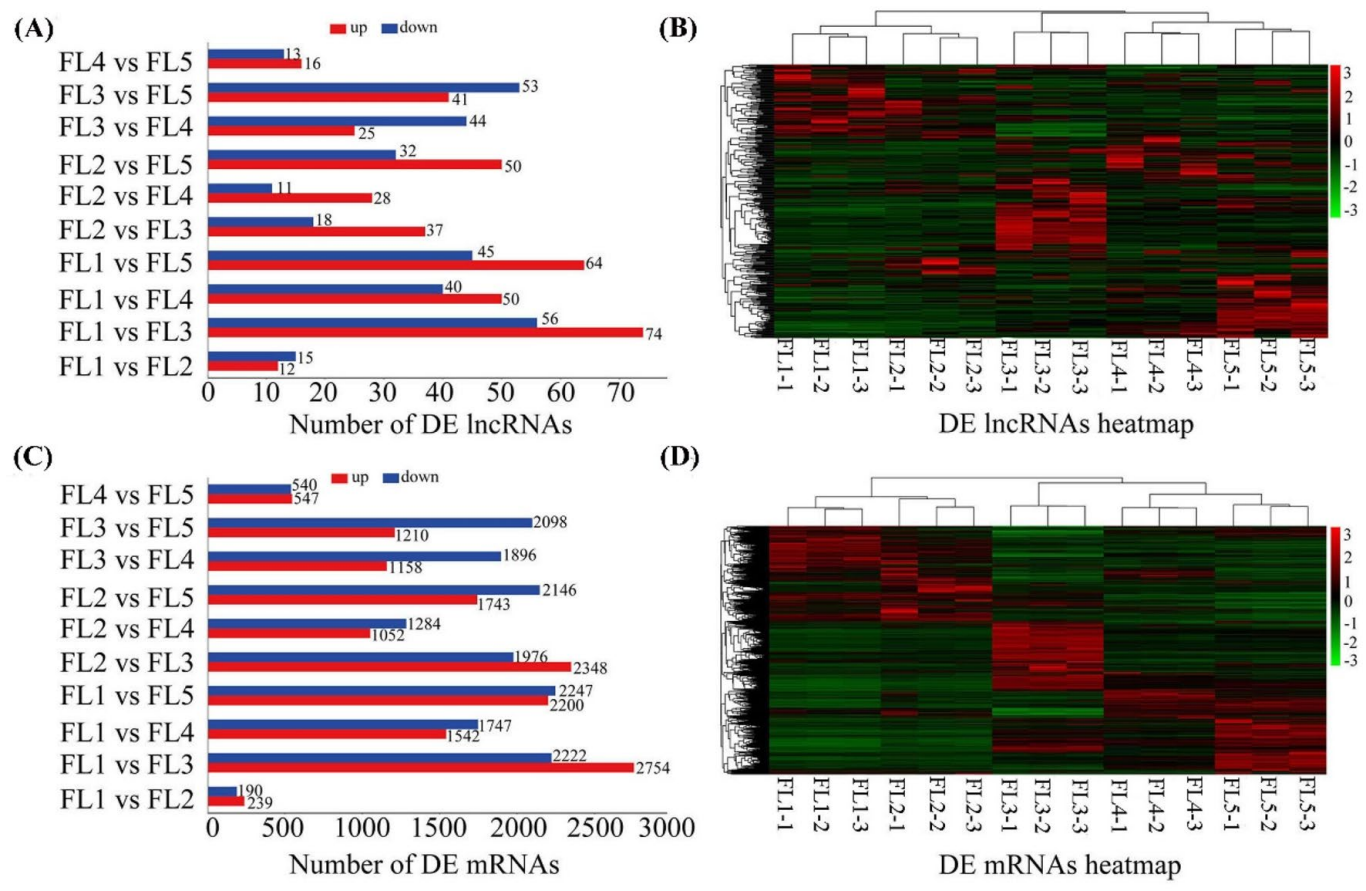
The identification and hierarchical cluster analysis of the DE lncRNAs and DE mRNAs. **a** The number of identified DE lncRNAs in each pairwise comparison group. **b** Hierarchical cluster analysis of the total DE lncRNAs. **c** The number of identified DE mRNAs in each pairwise comparison group. **d** Hierarchical cluster analysis of the total DE mRNAs

To explore the transcriptional changes of lncRNAs and mRNAs at five stages, a total of ten pairwise comparisons groups were analyzed in this study. Based on the screening criteria of log2 (fold change) > 1 and false discovery rate < 0.05, 27 (FL1 vs FL2), 130 (FL1 vs FL3), 90 (FL1 vs FL4), 109 (FL1 vs FL5), 55 (FL2 vs FL3), 39 (FL2 vs FL4), 82 (FL2 vs FL5), 69 (FL3 vs FL4), 94 (FL3 vs FL5), and 29 (FL4 vs FL5) DE lncRNAs were respectively identified in these ten groups (Fig. 3a, Table S5). Meanwhile, 429 (FL1 vs FL2), 4976 (FL1 vs FL3), 3289 (FL1 vs FL4), 4447 (FL1 vs FL5), 4324 (FL2 vs FL3), 2336 (FL2 vs FL4), 3889 (FL2 vs FL5), 3054 (FL3 vs FL4), 3308 (FL3 vs FL5), and 1087 (FL4 vs FL5) DE mRNAs were respectively identified (Fig. 3c, Table S6). And the number of up- and down-regulated DE RNAs in each group were also displayed (Fig. 3a, c). For example, 56 DE lncRNAs and 2222 DE mRNAs were down-regulated while 74 DE lncRNAs and 2754 DE mRNAs were up-regulated in FL1 vs FL3 group. The largest number of DE RNAs in FL1 vs FL3 group, while very less DE RNAs were found in FL1 vs FL2 and FL4 vs FL5 groups (Fig. 3a, c). Eventually, a total of 343 DE lncRNAs and 9412 DE mRNAs were obtained (Tables S5 and S6). In addition, the hierarchical cluster analysis of DE lncRNAs and DE mRNAs suggested that the existing differences of expression patterns at five stages during the process of flag leaf development to senescence in rice (Fig. 3b, d).

### Analysis on potential target genes of lncRNAs

**Fig. 4:**
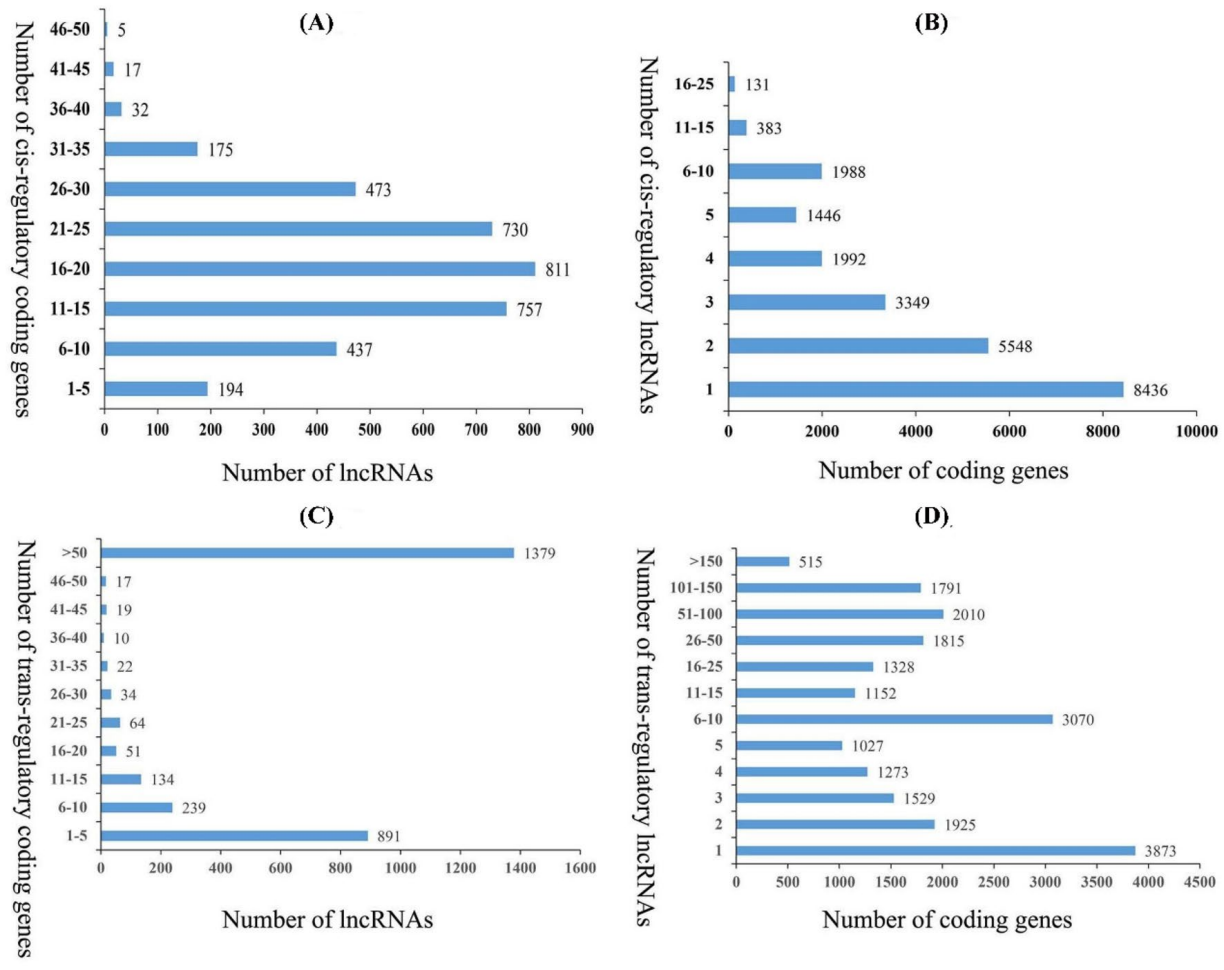
Analysis of cis-and trans-target genes of lncRNAs. **a** The number of cis-target genes regulated by lncRNAs. **b** The number of lncRNAs that have potential cis-regulatory effects on protein-coding genes. **c** The number of trans-target genes regulated by lncRNAs. **d** The number of lncRNAs that have potential trans-regulatory effects on protein-coding genes

LncRNAs have been found to regulate the expression of protein-coding genes through *cis*-and *trans*-acting models (Zhu et al. 2015). The protein-coding genes located within a genomic window of 100 kb upstream and downstream of lncRNAs were searched as potential *cis*-regulated target genes of lncRNAs (Tian et al. 2019). In this study, a total of 3631 lncRNAs were predicted to have potential *cis*-regulatory effects on 23273 protein-coding genes (Table S7). Among them, more than 80% lncRNAs targeted ten to fifty protein coding genes, and 22 lncRNAs have more than 40 target genes (Fig. 4a). More than 90% protein-coding genes corresponded to one to ten lncRNAs, and 514 protein-coding genes were predicted to be *cis*-regulated by more than ten lncRNAs (Fig. 4b). Moreover, lncRNAs may act in *trans*-, and the prediction of *trans*-regulation relies on the expression correlation between lncRNAs and mRNAs (Tian et al. 2019). In this study, a total of 2878 lncRNAs and 21307 associated target protein-coding genes were predicted to be *trans*-regulated (Table S7). Among these lncRNAs, 48% lncRNAs targeted more than 50 protein coding genes (Fig. 4c). Moreover, among the 21307 target protein-coding genes, 3873 (18%) corresponded to only one lncRNAs and up to 515 coding-genes were predicted to be targeted by more than 150 lncRNAs (Fig. 4d). These results may indicate the complex regulation relationships between lncRNAs and mRNAs.

### Analysis of lncRNAs co-expressed with mRNAs by WGCNA

**Fig. 5:**
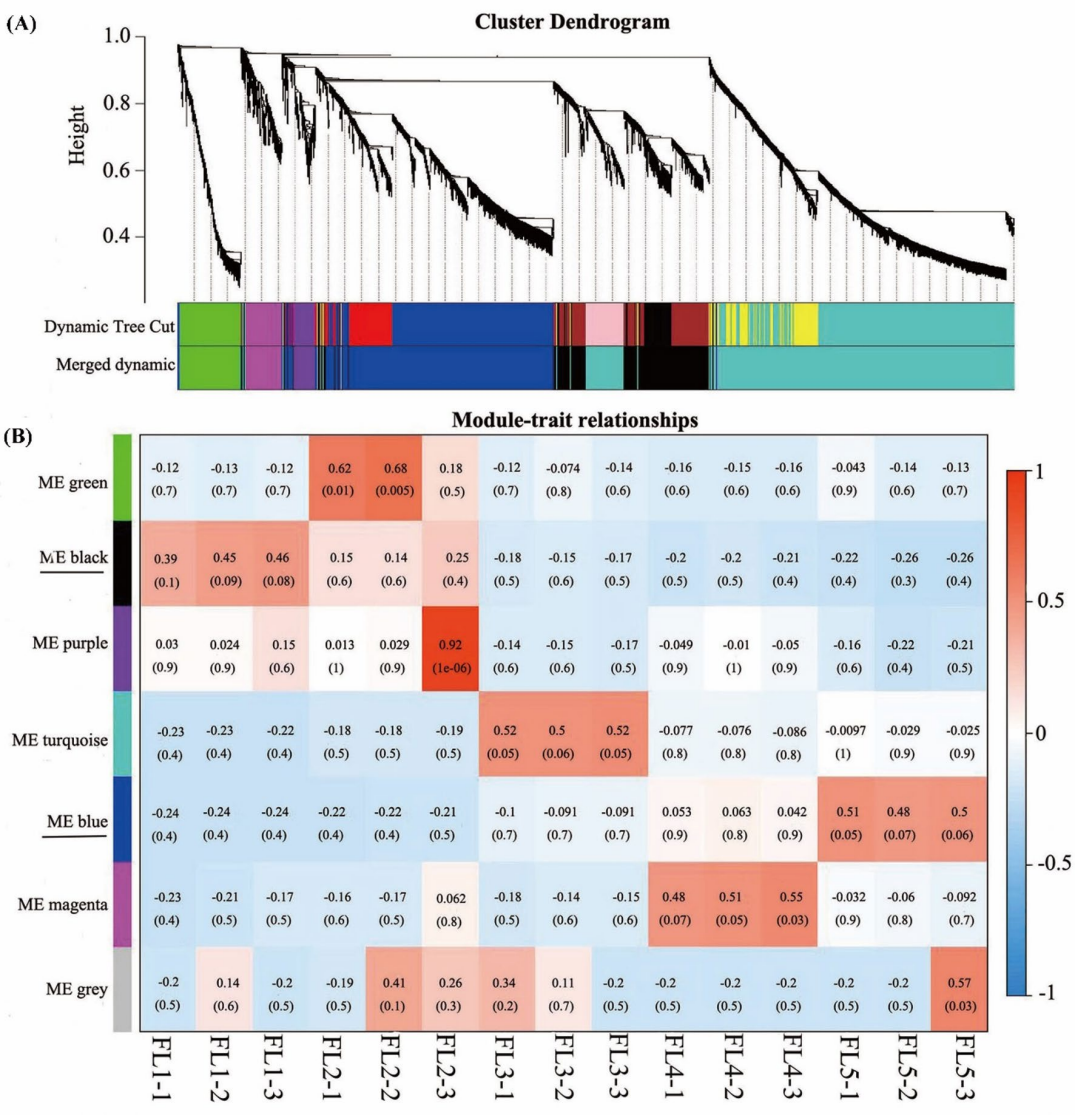
WGCNA of transcripts including DE lncRNAs and DE mRNAs involved in flag leaf senescence of rice. **a** Hierarchical cluster tree indicating seven modules identified by WGCNA. Each leaf in the tree is one gene and the major tree branches constitute seven modules labeled by different colors. **b** Module–trait correlations and corresponding p-values. Each row corresponds to a module and each column corresponds to a specific-stage sample. Each cell at the row–column intersection is color-coded by correlation according to the color legend

**Fig. 6:**
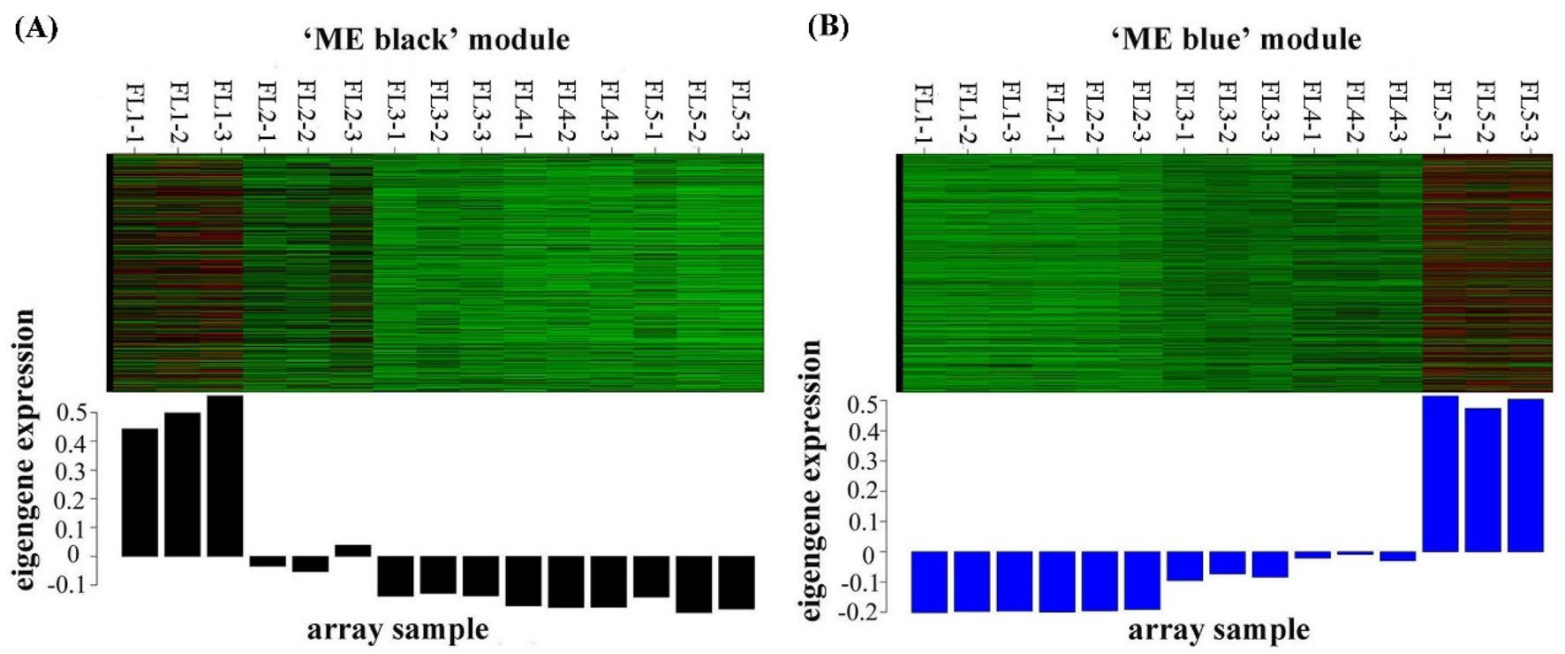
Co-expression network analysis of ‘ME black’ and ‘ME blue’ module in WGCNA. **a** Heatmap indicating the eigengene expression profile for the ‘ME black’ module. **b** Heatmap indicating the eigengene expression profile for the ‘ME blue’ module

To systematically explore the potential regulation functions of lncRNAs associated with flag leaf senescence, WGCNA was performed to analyze the co-expression relationship between 343 DE lncRNA transcripts and 9412 DE mRNA transcripts. A total of seven distinct modules were obtained, in which major tree branches define the modules (labeled with different colors), as shown in the dendrogram (Fig. 5a). Furthermore, the modules closely related to flag leaf senescence were of particular interest to investigate by analyzing the module-trait correlations. Notably, of the seven modules, the ‘ME black’ module displayed a continuous down-regulation trends accompanying with the flag leaf development from normal to senescence, whereas the ‘ME blue’ module showed a continuous up-regulation trends during this process of leaf senescence (Fig. 5b). And the heatmaps of the ‘ME black’ module and the ‘ME blue’ module showed the comprised transcripts which were the most highest expressed separately at booting stage (FL1) and at late senescence stage (FL5) (Fig. 6). Thus, considering the continuous down- and up-regulation trends during the process of flag leaf developing to senescence, the ‘ME black’ and ‘ME blue’ module were considered to be highly associated with leaf senescence (Fig. 5b).

The ‘ME black’ module comprised 22 DE lncRNA transcripts and 812 DE mRNA transcripts that displayed a continuous negative correlation with leaf senescence (Fig. 5b, Table S8). These 22 DE lncRNAs were predicted to target 2695 protein coding genes (Table S9), in which 378 target protein coding genes were DE mRNAs clustered in the ‘ME black’ module (Table S10). The ‘ME blue’ module comprised 48 DE lncRNA transcripts and 1209 DE mRNAs transcripts that displayed a continuous positive correlation with leaf senescence (Fig. 5b, Table S8). These 48 DE lncRNAs were predicted to target 3361 protein coding genes (Table S9), in which 941 target protein coding genes were DE mRNAs clustered in the ‘ME blue’ module (Table S10). The 22 DE lncRNAs from the ‘ME black’ module and 48 DE lncRNAs from the ‘ME blue’ module were selected as the most interested lncRNAs involving in flag leaf senescence.

### Enrichment analysis of co-expressed mRNAs targeted by interested lncRNAs involving in flag leaf senescence

**Fig. 7:**
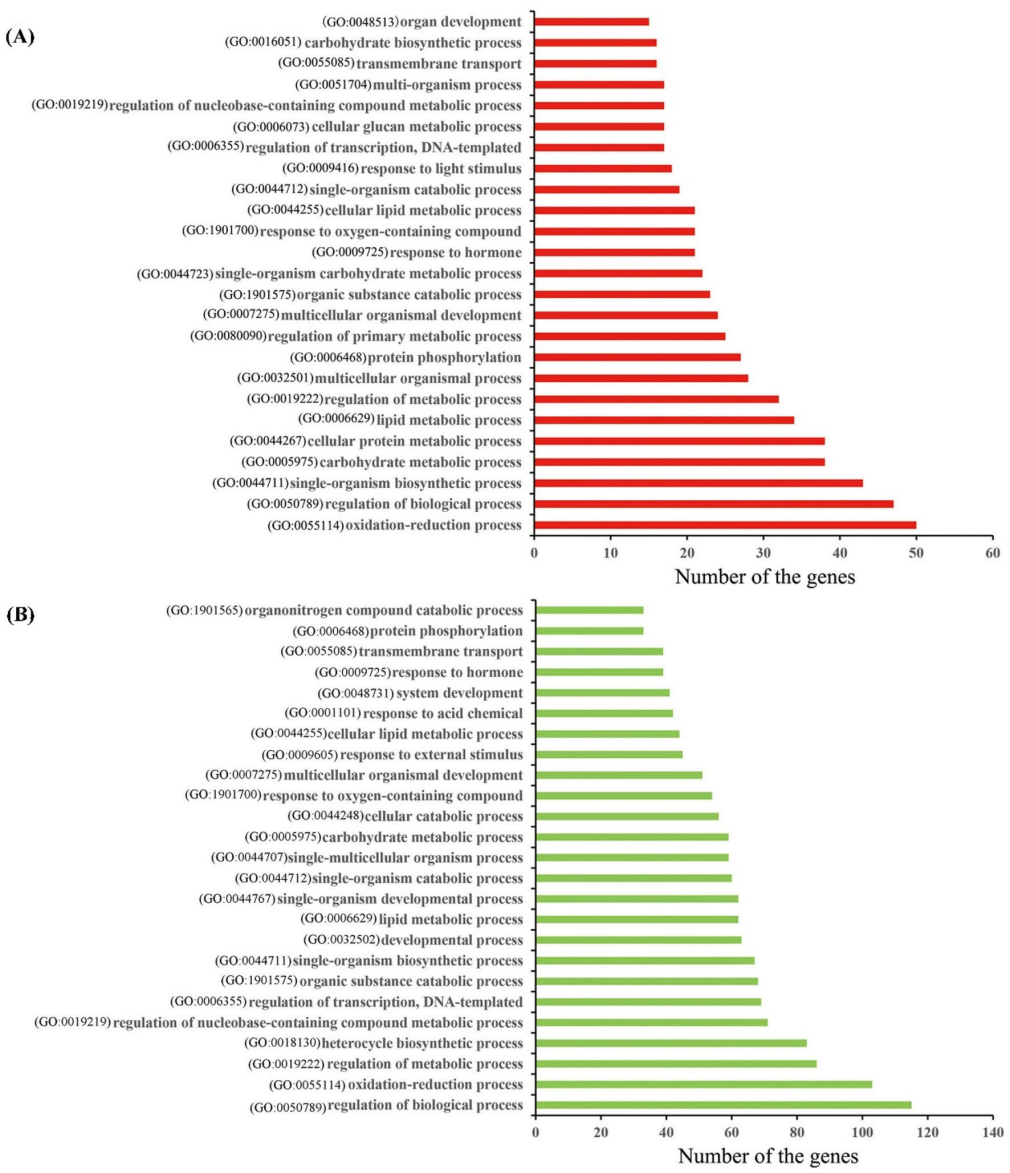
GO enrichment analysis on target DE mRNAs regulated by DE lncRNAs. **a** The top 25 GO terms for 378 target DE mRNAs regulated by 22 down-regulated DE lncRNAs in ‘ME black’ module. **b** The top 25 GO terms for 941 target DE mRNAs regulated by 48 up-regulated DE lncRNAs in ‘ME blue’ module

To investigate the potential functions of the interested lncRNAs involving in flag leaf senescence, GO enrichment analysis were separately performed for 378 co-expressed DE mRNAs targeted by 22 interested down-regulated lncRNAs in ‘ME black’ module and 941 co-expressed DE mRNAs targeted by 48 interested up-regulated lncRNAs in ‘ME blue’ module. For interested down-regulated lncRNAs, their co-expressed mRNAs were enriched in 485 GO terms (*P* < 0.05), among which, 343, 36, and 106 were classified under biological process, cellular component and molecular function, respectively (Table S11). Meanwhile, for interested up-regulated lncRNAs, their co-expressed mRNAs were enriched in 629 GO terms (*P* < 0.05), among which, 443, 45, and 141 were classified under biological process, cellular component and molecular function, respectively (Table S11). Twenty-five significant top GO terms of biological process were additionally listed (Fig. 7). Results showed that there were a lot of GO terms both found for co-expressed mRNAs targeted by down-regulated and up-regulated lncRNAs, including ‘oxidation–reduction process (GO:0055114)’, ‘regulation of biological process (GO:0050789)’, ‘single-organism biosynthetic process (GO:0044711)’, ‘carbohydrate metabolic process (GO:0005975)’, ‘lipid metabolic process (GO:0006629)’, ‘regulation of metabolic process (GO:0019222)’, ‘protein phosphorylation (GO:0006468)’, ‘multicellular organismal development (GO:0007275)’, ‘organic substance catabolic process (GO:1901575)’, ‘response to hormone (GO:0009725)’, ‘response to oxygen-containing compound (GO:1901700)’, ‘cellular lipid metabolic process (GO:0044255)’, ‘single-organism catabolic process (GO:0044712)’, ‘regulation of transcription, DNA-templated (GO:0006355)’, ‘regulation of nucleobase-containing compound metabolic process (GO:0019219)’ and ‘transmembrane transport (GO:0055085)’. These findings suggested that the senescence negatively and positively associated DE lncRNAs targeted DE mRNAs involving in many similar or common biological processes, including transcription, hormone response, oxidation–reduction process and substance metabolism.

**Fig. 8:**
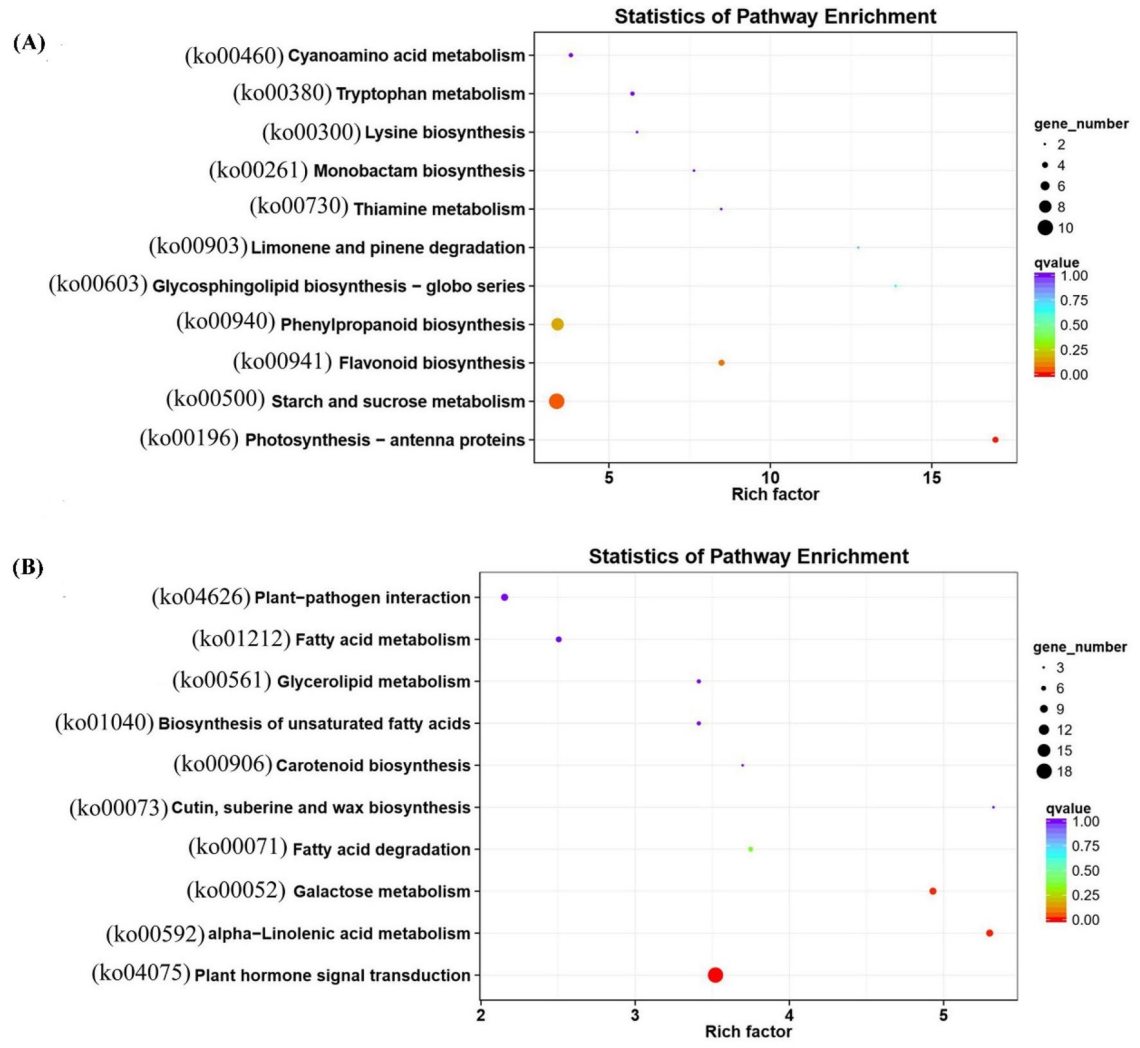
KEGG enrichment analysis on target DE mRNAs regulated by DE lncRNAs. **a** The KEGG enrichment analysis on 378 target DE mRNAs regulated by 22 down-regulated DE lncRNAs in ‘ME black’ module. **b** The KEGG enrichment analysis on 941 target DE mRNAs regulated by 48 up-regulated DE lncRNAs in ‘ME blue’ module

Moreover, statistical enrichment of KEGG was analyzed for the co-expressed mRNAs targeted by interested DE lncRNAs involving in flag leaf senescence. Results showed that the co-expressed mRNAs targeted by down-regulated lncRNAs were significantly enriched in 11 KEGG pathways, in which ‘photosynthesis—antenna proteins (ko00196)’ and ‘starch and sucrose metabolism (ko00500)’ were the most enriched (Fig. 8a, Table S12). Meanwhile, the co-expressed mRNAs targeted by up-regulated lncRNAs were significantly enriched in 10 KEGG pathways, in which ‘plant hormone signal transduction (ko04075)’, ‘alpha-Linolenic acid metabolism (ko00592)’ and ‘galactose metabolism (ko00052)’ were the most enriched (Fig. 8b, Table S12).

### Identification of interested lncRNAs targeting transcription factors (TFs)

It has been demonstrated that transcription factors (TFs) could play essential roles in senescence process (Podzimska-Sroka et al. 2015). In this study, a total of 378 target DE mRNAs targeted by 22 down-regulated DE lncRNA in the ‘ME black’ module were submitted to the Plant Transcription Factor Database (http://planttfdb.cbi.pku.edu.cn/). Totally, 25 DE mRNAs were found to encode TFs, which were targeted by 10 DE lncRNAs in ‘ME black’ module. (Table S13). For example, the gene BGIOSGA002217 encoding a C3H transcription factor was the target gene of lncRNA MSTRG.1416.1. Interestingly, one lncRNA could regulate multiple TFs, and one TF could also be regulated by multiple lncRNAs. For example, lncRNA MSTRG.56765.1 was predicted to regulate three TFs, containing BGIOSGA001872 (MYB family), BGIOSGA027617 (LSD family) and BGIOSGA029710 (C2H2 family) (Table S13). And the NAC transcription factor BGIOSGA036980 was predicted to be regulated by three lncRNAs MSTRG.30264.1, MSTRG.24808.2 and MSTRG.65878.1 (Table S13). Similarly, a total of 941 DE mRNAs targeted by 48 up-regulated DE lncRNAs in the ‘ME blue’ module were also searched in the Plant Transcription Factor Database. And 86 DE mRNAs were found to encode TFs, which were predicted to be the target genes of 33 DE lncRNAs (Table S13). The complex regulatory relationship between these DE lncRNAs and their target TFs in ‘ME blue’ module were also found. For example, lncRNA MSTRG.12677.1 was predicted to regulate 47 TFs, such as BGIOSGA000374 (NAC family), BGIOSGA013831 (C2H2 family) and BGIOSGA023457 (NAC family) (Table S13). And the NAC transcriptional factor BGIOSGA000374 was predicted to be regulated by 14 lncRNAs, such as MSTRG.12677.1, MSTRG.30528.1 and MSTRG.74673.3 (Table S13). These results suggested that senescence associated DE lncRNAs might play roles in leaf senescence through regulating transcriptional factors.

### Identification of lncRNAs targeting well-studied senescence-associated genes (SAGs)

So far, a vast number of senescence-associated genes (SAGs) were identified to play roles in leaf senescence. It is interesting to investigate whether these well-studied SAGs were regulated by the identified senescence-associated lncRNAs in this study. Results showed that a total of eight down-regulated DE lncRNAs in the ‘ME black’ module were predicted to target 12 identified SAGs, of which three SAGs were in the ‘ME black’ module while the other nine were not (Table 2). For example, the DE lncRNA MSTRG.1416.1 targeted the DE mRNA BGIOSGA002217 (*OsDOS*) that was clustered in the ‘ME black’ module, while two DE lncRNAs (MSTRG.24808.2, MSTRG.65878.1) were found to target the mRNA BGIOSGA028186 (*SPL29*) which was absent in the ‘ME black’ module (Table 2). Similarly, a total of 29 up-regulated DE lncRNAs in the ‘ME blue’ module were found to target 20 identified SAGs, in which 13 identified SAGs were found to locate in the same ‘ME blue’ module while the other seven SAGs were not (Table 2). For example, DE lncRNA MSTRG.8133.1 and its targeted mRNA BGIOSGA004591 (*OsPME1*) were both clustered in the ‘ME blue’ module, while BGIOSGA012281 (*PLS2*) targeted by the DE lncRNA MSTRG.20126.1, was not located in the ‘ME blue’ module. Interestingly, it was apparent to find that one identified SAG could be targeted by multiple lncRNAs. For example, the mRNA BGIOSGA023457 (*ONAC011*) was the target gene of 21 DE lncRNAs (Table 2). Meanwhile, one lncRNA could also be found to target multiple identified SAGs (Table S14). For instance, the DE lncRNA MSTRG.24808.2 in the ‘ME black’ module was found to target BGIOSGA004283 (*SPL28*), BGIOSGA028186 (*SPL29*), BGIOSGA033278 (*OsGDCH*) and BGIOSGA014571 (*cZOGT2*), while MSTRG.77321.3 in the ‘ME blue’ module could target multiple SAGs such as BGIOSGA023457 (*ONAC011*), BGIOSGA011859 (*OsPAO*) and BGIOSGA011953 (*OsFBK12*) (Table S14). Eventually, the expression levels of down-expressed lncRNA MSTRG.24808.2 and its targeted mRNAs (BGIOSGA033278 and BGIOSGA014571), and that of up-expressed lncRNA MSTRG.77321.3 and its targeted mRNAs (BGIOSGA023457 and BGIOSGA011859) were verified by qRT-PCR (Fig. 9). These results together implied that the senescence-associated lncRNAs played important roles in leaf senescence by regulating numerous senescence-associated mRNA transcripts. And these results also supplied evidences for the involvement of identified lncRNAs in flag leaf senescence.

**Table 2:** The identified well-studied senescence-associated genes targeted by lncRNAs

#lncRNA ID	lncRNA module	mRNA ID	mRNA module	RAP ID	Gene name	Phenotype			References
						Mutant	Over expression	Knockout	
MSTRG.1416.1	Black	BGIOSGA002217	Black	*Os01g0192000*	*OsDOS*	**/**	Delayed	RNAi: accelerated	Kong et al. (2006)
MSTRG.5543.1	Black	BGIOSGA019953	/	*Os05g0449500*	*OsCOI1b*	Stay green	/	CRISPR/Cas9:delayed	Lee et al. (2015)
MSTRG.24808.2/MSTRG.65878.1	Black	BGIOSGA014571	Black	*Os04g0556550*	*cZOGT2*	*/*	Delayed	/	Kudo et al. (2012)
MSTRG.56765.1	Black	BGIOSGA020059	/	*Os05g0481000*	*OsSNAT1*	/	Delayed	/	Lee and Back (2017)
MSTRG.65878.1	Black	BGIOSGA026526	/	*Os08g0549100*	*OsAPX4*	/	/	RNAi: accelerated	Ribeiro et al. (2017
MSTRG.44302.1	Black	BGIOSGA013898	/	*Os03g0837300*	*OsNaPRT1*	Early	Delayed	/	Wu et al. (2016)
MSTRG.5543.1	Black	BGIOSGA022234	/	*Os06g0130000*	*SPL4*	Early	/	T-DNA: delayed	Song et al. (2019)
MSTRG.24808.2/MSTRG.65878.1	Black	BGIOSGA004283	/	*Os01g0703600*	*SPL28*	Early	/	/	Qiao et al. (2010)
MSTRG.24808.2/MSTRG.65878.1	Black	BGIOSGA028186	/	*Os08g0206900*	*SPL29*	Early	/	/	Wang et al. (2015)
MSTRG.65878.1	Black	BGIOSGA020739	/	*Os06g0662000*	*OsPLS1*	Early		/	Yang et al. (2016)
MSTRG.51442.2	Black	BGIOSGA014554	/	*Os04g0560600*	*OsCPK12*	Early	Delayed	CRISPR/Cas9:accelerated	Wang et al. (2019b)
MSTRG.44376.1/MSTRG.24808.2/MSTRG.65878.1	Black	BGIOSGA033278	Black	*Os10g0516100*	*OsGDCH*	/	/	RNAi: accelerated	Zhou et al. (2013)
MSTRG.12677.1/MSTRG.79278.1/MSTRG.74673.1/MSTRG.74673.4/MSTRG.30528.1/MSTRG.43455.1/MSTRG.7519.1/MSTRG.74673.3/MSTRG.80924.2/MSTRG.29832.1/MSTRG.77321.3/MSTRG.19268.3/MSTRG.42965.1/MSTRG.12677.3/MSTRG.76387.1/MSTRG.552.1/MSTRG.84624.1/MSTRG.8133.1/MSTRG.8050.1/MSTRG.4640.1/MSTRG.4077.5	Blue	BGIOSGA023457	Blue	*Os06g0675600*	*ONAC011*	/	Accelerated	RNAi: delayed	El Mannai et al. (2017)
MSTRG.79278.1/MSTRG.57335.8/MSTRG.26261.1/MSTRG.19268.3/MSTRG.76387.1/MSTRG.552.1/MSTRG.84624.1	Blue	BGIOSGA019953	/	*Os05g0449500*	*OsCOI1b*	Stay green	/	CRISPR/Cas9:delayed	Lee et al. (2015)
MSTRG.8133.1	Blue	BGIOSGA004591	Blue	*Os01g0788400*	*OsPME1*	/	Accelerated	RNAi: delayed	Fang et al. (2016)
MSTRG.12677.1/MSTRG.79278.1/MSTRG.57335.8/MSTRG.74673.4/MSTRG.11508.1/MSTRG.74673.3/MSTRG.29832.1/MSTRG.77321.3/MSTRG.19268.3/MSTRG.12677.3/MSTRG.76387.1/MSTRG.552.1	Blue	BGIOSGA013214	Blue	*Os03g0645900*	*OsNCED3*	/	Accelerated	CRISPR/Cas9:delayed	Huang et al. (2018)
MSTRG.4640.1/MSTRG.4077.5	Blue	BGIOSGA004316	Blue	*Os01g0711600*	*OsRTH1*	/	Delayed	/	Zhang et al. (2012)
MSTRG.79278.1	Blue	BGIOSGA026526	/	*Os08g0549100*	*OsAPX4*	/	/	RNAi: accelerated	Ribeiro et al. (2017)
MSTRG.12677.1/MSTRG.79278.1/MSTRG.74673.1/MSTRG.74673.4/MSTRG.30528.1/MSTRG.43455.1/MSTRG.7519.1/MSTRG.74673.3/MSTRG.80924.2/MSTRG.29832.1/MSTRG.77321.3/MSTRG.19268.3/MSTRG.42965.1/MSTRG.12677.3/MSTRG.76387.1/MSTRG.552.1/MSTRG.84624.1/MSTRG.8133.1/MSTRG.8050.1/MSTRG.4640.1/MSTRG.4077.5	blue	BGIOSGA003046	blue	*Os01g0227100*	*NYC1*	Stay green	/	/	Kusaba et al. (2007)
MSTRG.12677.1/MSTRG.79278.1/MSTRG.74673.4/MSTRG.43455.1/MSTRG.11508.1/MSTRG.7519.1/MSTRG.74673.3/MSTRG.80924.2/MSTRG.29832.1/MSTRG.77321.3/MSTRG.19268.3/MSTRG.42965.1/MSTRG.12677.3/MSTRG.76387.1/MSTRG.552.1/MSTRG.53258.3/MSTRG.8050.1/MSTRG.4077.5	Blue	BGIOSGA010125	Blue	*Os03g0654600*	*NOL*	Stay green	/	/	Sato et al. (2009)
MSTRG.19268.3/MSTRG.76387.1/MSTRG.552.1	Blue	BGIOSGA010917	/	*Os04g0320100*	*OsHCAR*	persistent green	/	/	Piao et al. (2017)
MSTRG.12677.1/MSTRG.79278.1/MSTRG.57335.8/MSTRG.29832.1/MSTRG.77321.3/MSTRG.19268.3/MSTRG.12677.3/MSTRG.76387.1/MSTRG.552.1/MSTRG.39310.1/MSTRG.84624.1	Blue	BGIOSGA011859	Blue	*Os03g0146400*	*OsPAO*	/	/	RNAi: death	Tang et al. (2011)
MSTRG.12677.1/MSTRG.79278.1/MSTRG.57335.8/MSTRG.29832.1/MSTRG.77321.3/MSTRG.26261.1/MSTRG.19268.3/MSTRG.12677.3/MSTRG.76387.1/MSTRG.552.1/MSTRG.39310.1/MSTRG.84624.1	Blue	BGIOSGA032012	Blue	*Os10g0389200*	*OsRCCR1*	/	/	RNAi: death	Tang et al. (2011)
MSTRG.57335.8/MSTRG.26261.1/MSTRG.19268.3/MSTRG.76387.1/MSTRG.552.1/MSTRG.39310.1/MSTRG.84624.1	Blue	BGIOSGA022884	Blue	*Os06g0354700*	*NYC3*	Stay green	/	/	Morita et al. (2009)
MSTRG.12677.3	Blue	BGIOSGA024095	/	*Os07g0558500*	*NYC4*	Stay green	/	/	Yamatani et al. (2013)
MSTRG.12677.1/MSTRG.79278.1/MSTRG.57335.8/MSTRG.74673.4/MSTRG.43455.1/MSTRG.11508.1/MSTRG.74673.3/MSTRG.29832.1/MSTRG.77321.3/MSTRG.19268.3/MSTRG.12677.3/MSTRG.76387.1/MSTRG.552.1/MSTRG.53258.3/MSTRG.8133.1/MSTRG.8050.1/MSTRG.4077.5	Blue	BGIOSGA029383	Blue	*Os09g0532000*	*SGRL*	/	Accelerated	/	Rong et al. (2013)
MSTRG.54054.1	Blue	BGIOSGA028915	Blue	*Os08g0495800*	*OsAkαGa*	Delayed		/	Lee et al. (2009)
MSTRG.20126.1/MSTRG.76387.1/MSTRG.39310.1/MSTRG.84624.1/MSTRG.13889.1	Blue	BGIOSGA022234	/	*Os06g0130000*	*SPL4*	/	/	T-DNA: delayed	Song et al. (2019)
MSTRG.39310.1/MSTRG.84624.1/MSTRG.8133.1	Blue	BGIOSGA020739	/	*Os06g0662000*	*OsPLS1*	Early	/	/	Yang et al. (2016)
MSTRG.20126.1	Blue	BGIOSGA012281	/	*Os03g0265100*	*PLS2*	Early	Delayed	CRISPR/Cas9:accelerated	Wang et al. (2018a, b)
MSTRG.12677.1/MSTRG.74673.1/MSTRG.29832.1/MSTRG.77321.3/MSTRG.19268.3/MSTRG.42965.1/MSTRG.552.1/MSTRG.8050.1/MSTRG.4640.1/MSTRG.4077.5	Blue	BGIOSGA001700	Blue	*Os01g0348900*	*salT*	/	Delayed	/	Zhu et al. (2019)
MSTRG.12677.1/MSTRG.30528.1/MSTRG.29832.1/MSTRG.77321.3/MSTRG.19268.3/MSTRG.42965.1/MSTRG.20126.1/MSTRG.76387.1/MSTRG.552.1/MSTRG.39310.1/MSTRG.84624.1/MSTRG.8133.1/MSTRG.8050.1/MSTRG.4640.1/MSTRG.4077.5	Blue	BGIOSGA011953	Blue	*Os03g0171600*	*OsFBK12*	/	Delayed	RNAi: accelerated	Chen et al. (2013)

**Fig. 9:**
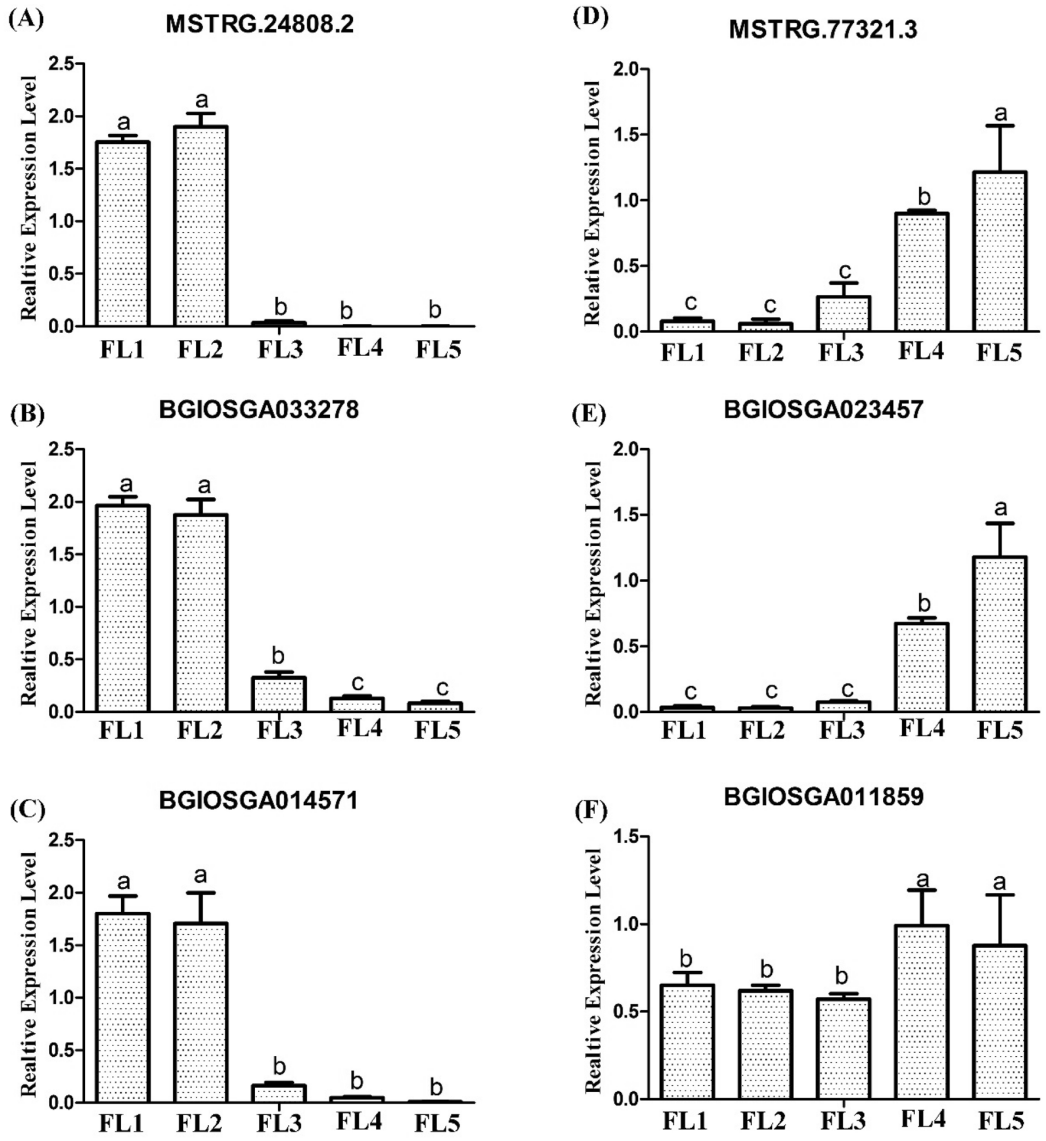
The qRT-PCR analysis of lncRNAs and their putative target genes. **a** lncRNA MSTRG.24808.2. **b** mRNA BGIOSGA033278. **c** BGIOSGA014571. **d** MSTRG.77321.3. **e** BGIOSGA023457. **f** BGIOSGA011859. The data are expressed as the mean ± SD of three biological replicates. Different letters indicate values are statistically different based on one-way ANOVA analysis

### Analysis of lncRNA-associated ceRNA network in senescent leaf of rice

It has been reported that lncRNAs can act as miRNA sponge to form a ceRNA network and thus regulate the expression of target transcripts of the same miRNAs (Cesana et al. 2011). In this study, two ceRNA networks were separately constructed using senescence associated DE lncRNAs and DE mRNAs from the ‘ME black’ module and ‘ME blue’ module, combining with miRNAs from miRBase (release 22, October 2018).

**Fig. 10:**
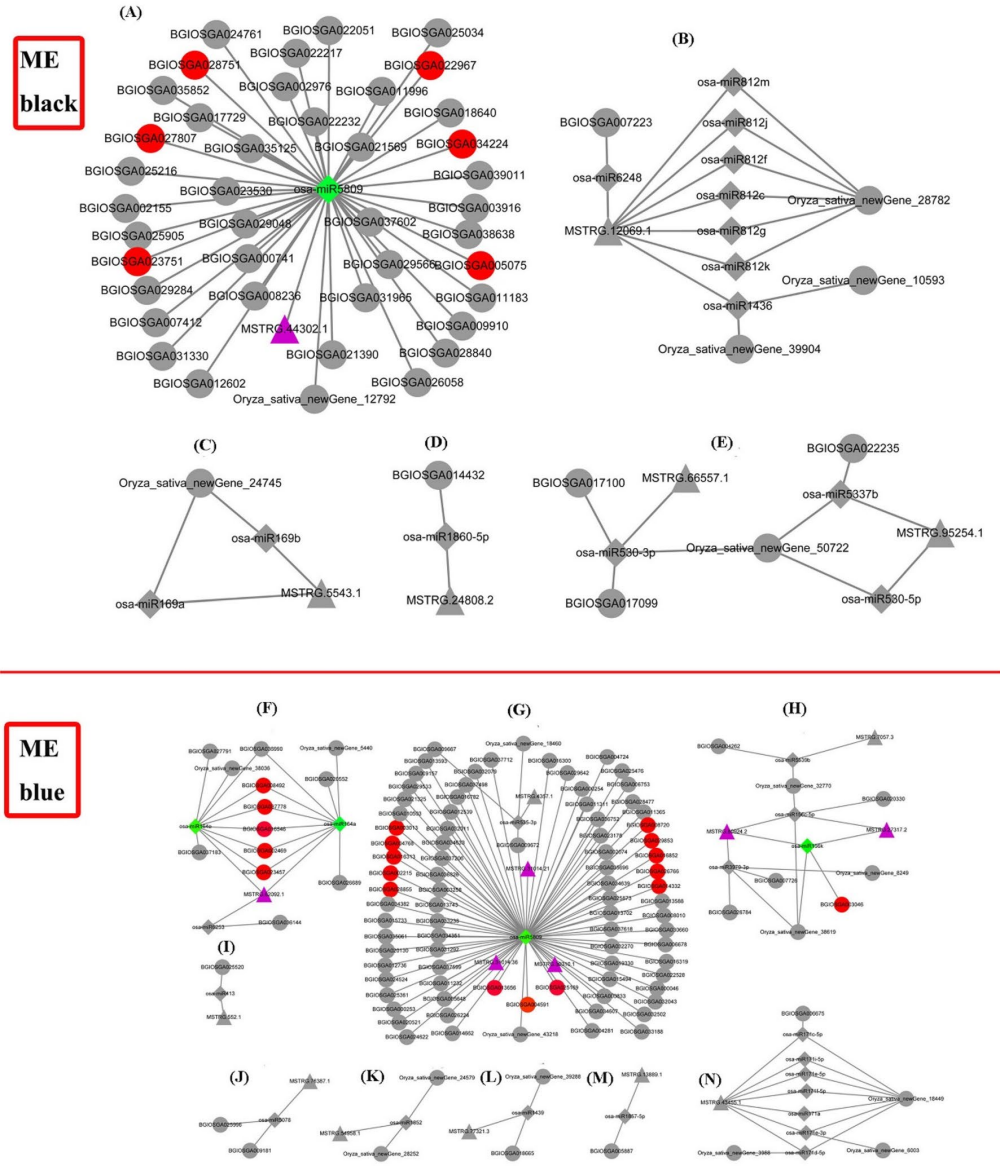
The lncRNAs-associated ceRNA networks formed by down- and up-regulated lncRNAs and their regulated co-expressed mRNAs. **a**–**e** The ceRNA subnetwork formed by down-regulated lncRNAs and their regulated co-expressed mRNAs in ‘ME black’ module. **f**–**n** The ceRNA subnetwork formed by up-regulated lncRNAs and their regulated co-expressed mRNAs in ‘ME blue’ module. Triangle, diamond and circle indicated the lncRNAs, miRNAs and mRNAs, respectively. Purple nodes, green nodes and red circle nodes represented the emphasized competitive lncRNAs, miRNAs and known mRNAs (transcription factors and known well-studied SAGs)

For down-regulated lncRNAs and their co-expressed mRNA in ‘ME black’ module, a total of five competitive relationship subnetworks were identified, including 6 lncRNAs, 15 miRNAs and 51 mRNAs (Fig. 10a–e). Among these 51 mRNAs, none was identified as well-studied SAG. However, it was interesting to found that one key lncRNA MSTRG.44302.1 (purple triangle) may bind with osa-miR5809 to regulate 41 protein coding genes, in which six mRNAs are TFs including BGIOSGA028751 (AP2 family), BGIOSGA005075 (SBP family), BGIOSGA022967 (bHLH family), BGIOSGA027807 (NAC family), BGIOSGA034224 (ZF-HD family) and BGIOSGA023751 (CO-like family) (Fig. 10a).

Accordingly, a total of nine competitive relationship sub-networks were identified for up-regulated lncRNAs and their co-expressed mRNA in ‘ME blue’ module, including 14 lncRNAs, 21 miRNAs and 117 mRNAs (Fig. 10f–n). Interestingly, some TFs and well-studied SAGs were found in networks. For example, one key lncRNA MSTRG.62092.1 may combine with osa-miR164a and osa-miR164e to regulate 12 protein coding genes, in which five mRNAs are TFs including BGIOSGA037778 (NAC family), BGIOSGA008492 (NAC family), BGIOSGA016546 (*OsNAC2*, NAC family), BGIOSGA023457 (*ONAC011*, NAC family) and BGIOSGA002469 (MYB family) (Fig. 10f). And three key lncRNAs MSTRG.31014.21, MSTRG.31014.36 and MSTRG.39310.1 may uniformly bind with osa-miR5809 to regulate 82 protein coding genes, in which three mRNAs are identified well-studied SAGs, including BGIOSGA013656 (*ONAC066*, NAC family), BGIOSGA004591 (*OsPME1*) and BGIOSGA025169 (*OsNCED4*), meanwhile, another 10 mRNAs were various TFs, containing BGIOSGA003013 (bHLH family), BGIOSGA004768 (C2H2 family), BGIOSGA016313 (ERF family), BGIOSGA002215 (MYB_related family), BGIOSGA028855 (bHLH family), BGIOSGA008720 (HD-ZIP family), BGIOSGA029853 (HD-ZIP family), BGIOSGA016852 (HD-ZIP family), BGIOSGA026766 (bZIP family), and BGIOSGA014332 (NAC family) (Fig. 10g). Moreover, lncRNAs MSTRG.80924.2 and MSTRG.27317.2 may bind osa-miR156k to regulate the chlorophyll-degradation associated gene BGIOSGA003046 (*NYC1*) (Fig. 10h).

**Fig. 11:**
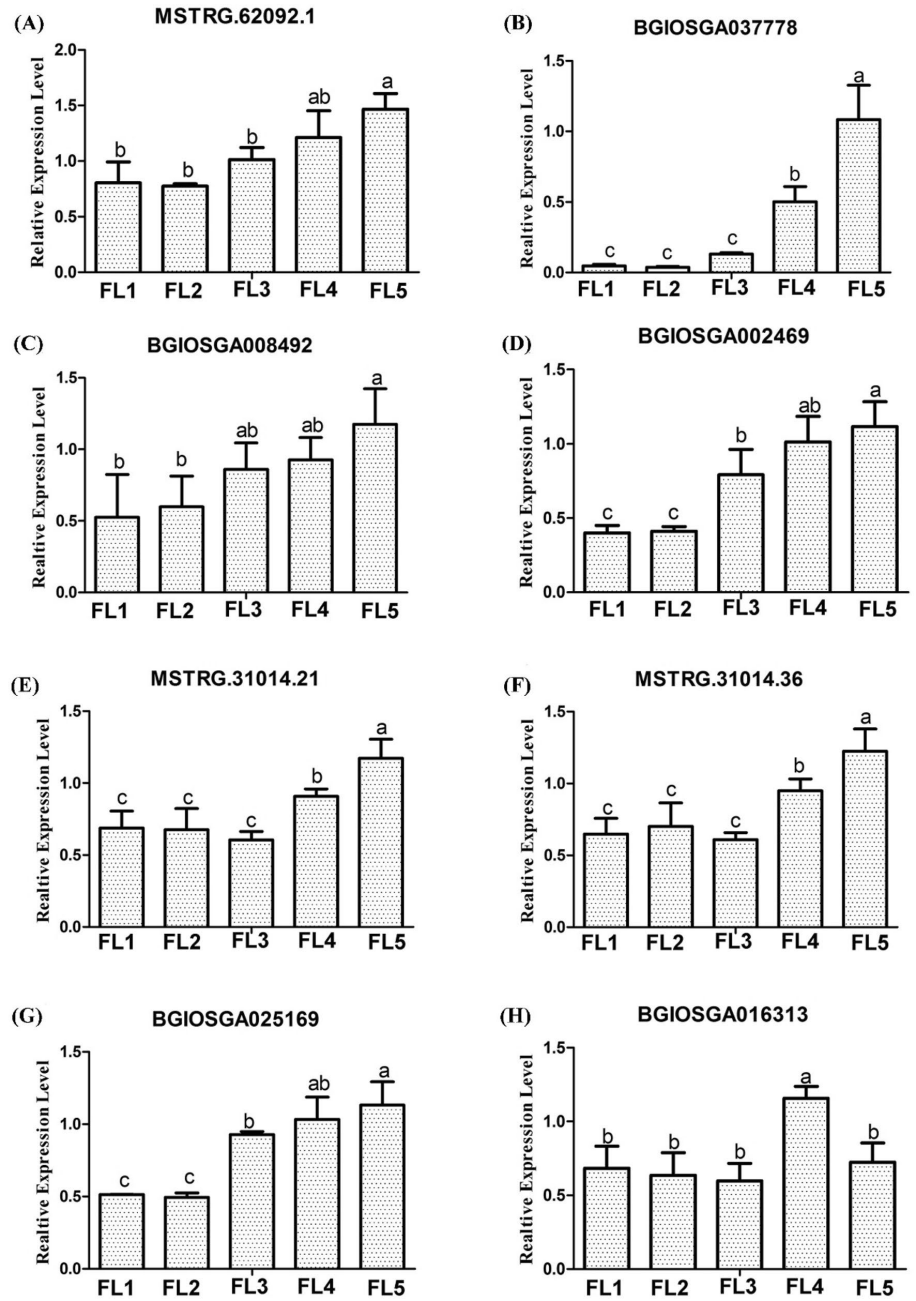
The qRT-PCR validation of DE lncRNAs and co-expressed mRNAs in ceRNA network. **a** lncRNA MSTRG.62092.1. **b** mRNA BGIOSGA037778. **c** mRNA BGIOSGA008492. **d** mRNA BGIOSGA002469. **e** lncRNA MSTRG.31014.21. **f** lncRNA MSTRG.31014.36. **g** mRNA BGIOSGA025169. **h** BGIOSGA016313. The data are expressed as the mean ± SD of three biological replicates. Different letters indicate values are statistically different based on one-way ANOVA analysis

To validate the regulatory relationship between lncRNAs and mRNAs, qRT-PCR experiments were performed. In the ceRNA network, five DE mRNAs and three DE lncRNAs were analyzed. The results showed that expression levels of lncRNA MSTRG.62092.1 and mRNAs BGIOSGA037778 (NAC family), BGIOSGA008492 (NAC family), and BGIOSGA002469 (MYB family) increased with the flag leaf developing to senescence (Fig. 11a–d), suggesting that lncRNA MSTRG.62092.1 could bind with osa-miR164a and osa-miR164e to regulate the expression of these three TFs in the leaf senescence process. Similarly, lncRNAs MSTRG.31014.21 and MSTRG.31014.36, and mRNA BGIOSGA025169 (*OsNCED4*) and BGIOSGA016313 (NAC family), also showed the increased expression levels during leaf senescence (Fig. 11e–h), implying that lncRNAs MSTRG.31014.21 and MSTRG.31014.36 could combine with miR5809 to regulate protein coding genes in this process. Thus, these key lncRNAs forming the ceRNA networks may play important functions in leaf senescence.

## Discussion

### LncRNA is a key regulator during flag leaf senescence in rice

LncRNAs were well demonstrated to play vital roles in various biological processes including gene silencing, abiotic stress response, growth and development (Liu et al. 2015b). To date, a vast number of lncRNAs were identified in plants, such as melon (Tian et al. 2019), cassava (Ding et al. 2019), Prunus mume (Wu et al. 2019), Arabidopsis (Liu et al. 2019), rice (Yu et al. 2020) and cotton (Deng et al. 2018), revealing their important roles in multiple different biological processes. However, no systematic identification of lncRNAs involving in leaf senescence were reported. In this study, a total of 3953 lncRNAs were identified by genome-wide high-throughput sequencing during the developmental process of rice flag leaf from normal to senescence, in which 343 lncRNAs were found to be differentially expressed (Table S5). Among all ten pairwise comparisons of five analyzed stages, group ‘FL1 vs FL3’ had the highest number of DE lncRNAs (Fig. 3a), implying that the senescence status of flag leaves changed significantly from booting stage (FL1) to early senescence stage (FL3). On the contrast, the senescence status was more uniform in flag leaves from booting stage (FL1) to flowering stage (FL2), and mid-senescence stage (FL4) to late-senescence stage (FL5). Furthermore, the continuous positive and negative modules (‘ME black’ and ‘ME blue’) correlated with leaf senescence were found by WGCNA analysis (Fig. 5), in which, DE lncRNAs targeted their co-expressed mRNAs participating in senescence-associated biological processes and pathways (Figs. 7 and 8). And these interested lncRNAs were also predicted to target transcription factors and well-studied SAGs (Tables 2 and S13). We speculated that lncRNAs in these two modules could most possibly play important roles in leaf senescence. In addition, the ceRNA network analysis revealed that some senescence-associated lncRNAs were predicted to regulate their co-expressed mRNAs through the miRNA sponges (Figs. 10 and 11). These results provided a molecular basis for understanding about regulatory functions of lncRNAs on flag leaf senescence, and laid a foundation for further functional studies on candidate lncRNAs.

### Involvement of lncRNAs in flag leaf senescence by targeting transcription factors

**Fig. 12:**
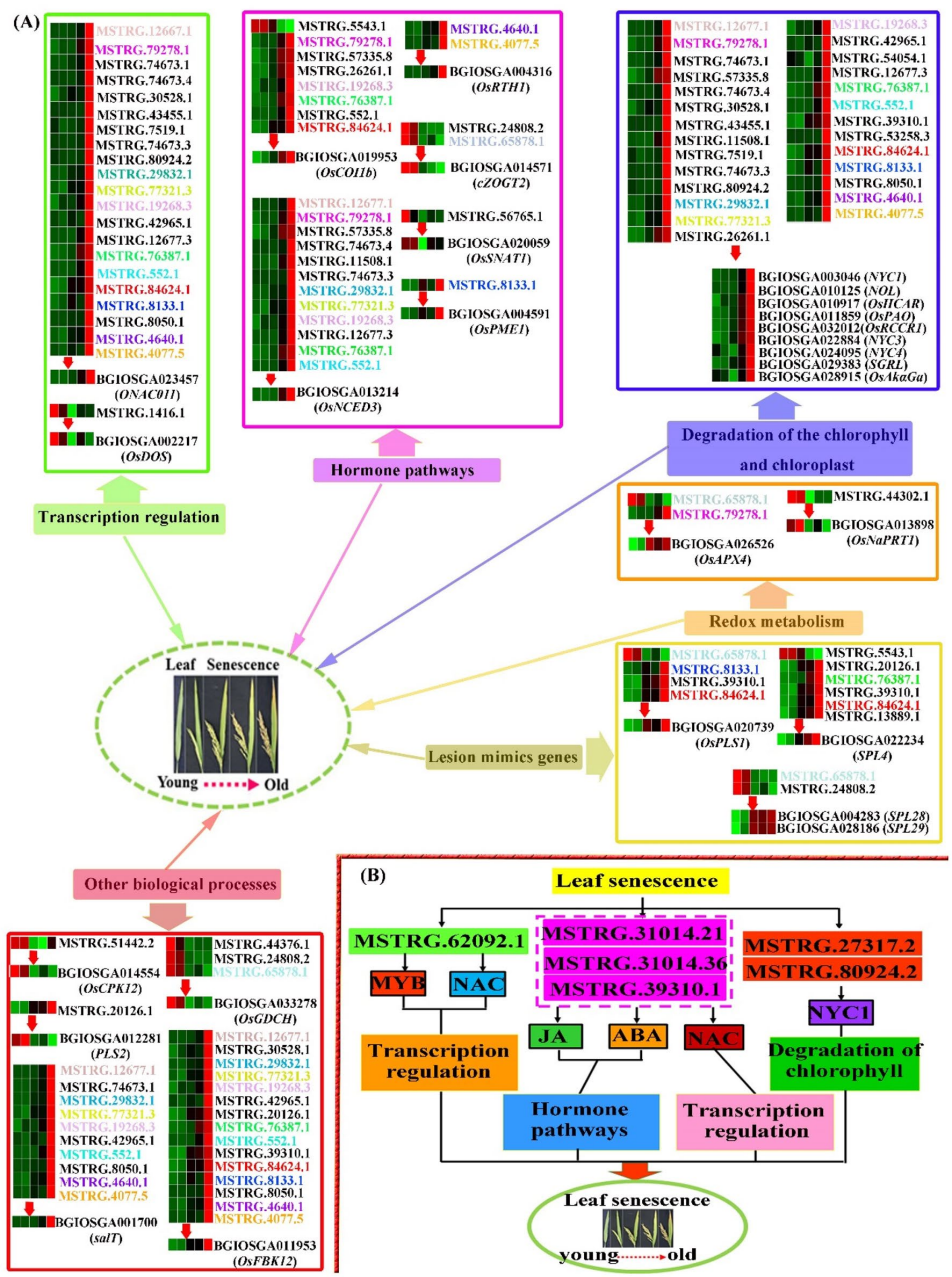
The network models of senescence-associated lncRNAs involved in flag leaf senescence. **a** A proposed regulatory mechanism involving lncRNAs and their potential and well-studied target genes during flag leaf senescence. The heatmaps represent the expression patterns of lncRNAs and their potential target genes in five analyzed leaf stages. LncRNAs participating in more than four different biological processes were labeled in different colors, and recommended to be prior candidates for transgenic analysis. **b** The model of lncRNAs by regulating well-studied mRNAs through ceRNA network to participate in senescence-related biological processes. LncRNAs in the dotted box probably regulating multiple senescence-related mRNAs, were recommended to be prior candidates for transgenic analysis

Transcription factors are important regulatory elements for the transcriptional activation or repression of target genes by recognizing their specific regulatory DNA sequences usually in the promotor region. Studies on the regulatory mechanisms and functions of transcription factors have increasingly been considered as research hotspots for leaf senescence (Podzimska-Sroka et al. 2015). In this study, whether senescence associated lncRNAs might regulate transcription factors were investigated. GO analysis revealed 17 and 69 mRNAs were separately enriched for down-regulated and up-regulated lncRNAs in the term of ‘regulation of transcription, DNA-templated (GO:0006355)’ (Fig. 7, Table S11). Additional analysis on transcription factors identified 25 and 86 transcription factors, which were separately targeted by 10 down-regulated lncRNAs and 33 up-expressed lncRNAs (Table S13). A complex regulation network might exist among these lncRNAs and TFs in the process of leaf senescence. Functional analysis of some specific TFs was reported for leaf senescence in rice. *OsDOS* is a novel nuclear-localized C3H-type transcription factor, and RNAi knockdown of *OsDOS* showed accelerated leaf senescence, shorter panicle and slightly reduced seed setting whereas its overexpression appeared a delayed leaf senescence and severe sterility (Kong et al. 2006). Gene BGIOSGA002217 was identified to be *OsDOS*, and it was predicted to be the target gene of lncRNA MSTRG.1416.1 (Table 2). *ONAC011* is a NAC-type transcription factor. Transgenic rice overexpressing *ONAC011* accelerated heading time and promoted leaf senescence, while blocking the function of *ONAC011* gene via RNA interference could delay both heading time and leaf senescence. Irrespective of early or delayed senescence, transgenic plants showed reduced grain yields (El Mannai et al. 2017). BGIOSGA023457 was identified to be *ONAC011*, and surprisingly, up to 21 DE lncRNAs were predicted to target *ONAC011* (Table 2). As a result, it is speculated that lncRNAs may play key roles in leaf senescence by transcription regulation (Fig. 12a).

### Involvement of lncRNAs in flag leaf senescence by targeting genes in hormone pathways

Leaf senescence is a developmental process controlled by complex networks of interacting genes and signaling pathways, and plant hormones play vital roles in regulating this process (Lim et al. 2007). In this study, during the process of investigating the functions of interested lncRNAs, a lot of hormone related GO terms were found, such as ‘response to hormone (GO:0009725)’, ‘hormone-mediated signaling pathway (GO:0009755)’, ‘hormone metabolic process (GO:0042445)’ (Fig. 7, Table S11). It is known to all that jasmonates act as a signaling in numerous developmental processes including senescence, which are formed from alpha-linolenic acid of chloroplast membranes by oxidative processes (Wasternack and Song 2016). Phenylpropanoid was reported to involve in the formation of salicylic acid, which played roles in leaf senescence (Strawn et al. 2007). And tryptophan was involved in the biosynthesis of melatonin, which could delay senescence and protect photosynthetic systems (Arnao and Hernández-Ruiz 2018). KEGG analysis revealed that ‘alpha-linolenic acid metabolism (ko00592)’, ‘phenylpropanoid biosynthesis (ko00940)’ and ‘tryptophan metabolism (ko00380)’ were all enriched in this study, and ‘plant hormone signal transduction (ko04075)’ was the most significantly enriched (Fig. 8, Table S12).

It is also founded that interested lncRNAs may target identified hormone-related genes involving in leaf senescence. In rice, mutation of *COI* homologs *OsCOI1b*, mainly affects leaf senescence by mediating jasmonate signaling, and further reduces grain production by decreasing spikelet fertility and grain weight (Lee et al. 2015). In this study, BGIOSGA019953 (*OsCOI1b*) was predicted to be the target gene of 7 lncRNAs (Table 2). *OsPME1* is an epigenetically regulated gene, affecting the content of jasmonates and regulating leaf senescence. Accelerated leaf senescence and chlorophyll degradation were observed in plants overexpressing *OsPME1*, while *OsPME1* RNAi plants displayed retarded leaf senescence (Fang et al. 2016). The gene BGIOSGA004591 (*OsPME1*) in ‘ME blue’ module was predicted to be the target gene of lncRNA MSTRG.8133.1 (Table 2). *OsNCED3* is a key gene in regulating abscisic acid biosynthesis, and overexpression of *OsNCED3* in transgenic rice could promote leaf senescence and increase abscisic acid content, whereas its knockout using CRISPR/Cas9 could delay leaf senescence (Huang et al. 2018). Gene BGIOSGA013214 in ‘ME blue’ module was identified to be *OsNCED3*, and this gene was predicted to be the target gene of 12 lncRNAs (Table 2). *OsRTH1* play roles in the modulation of ethylene responses, and its overexpression substantially prevented ethylene-induced alterations in growth and development, including leaf senescence (Zhang et al. 2012). Gene BGIOSGA004316 in ‘ME blue’ module was identified to be *OsRTH1*, and this gene was found to be the target gene of lncRNAs MSTRG.4640.1 and MSTRG.4077.5 (Table 2). Cytokinins was reported to involve in the regulation of various biological processes including senescence (Kim et al. 2006). The putative cis-Zeatin-*O*-glucosyltransferase gene *cZOGT2* plays roles in regulating cytokinin activity of cis-Zeatin, and transgenic rice lines ectopically overexpressing the *cZOGT2* gene exhibited delay of leaf senescence (Kudo et al. 2012). Interestingly, gene BGIOSGA014571 was found to be *cZOGT2*, and this gene was predicted to be the target gene of lncRNAs MSTRG.24808.2 and MSTRG.65878.1 (Table 2). Exogenous application of melatonin to rice plant indicated that melatonin acts as a biostimulator to delay leaf senescence (Liang et al. 2015). Serotonin *N*-acetyltransferase (SNAT) is a key gene for melatonin biosynthesis, and overexpression of *OsSNAT1* in transgenic rice plants confers resistance to cadmium and senescence, and increases grain yield (Lee and Back 2017). BGIOSGA020059 (*OsSNAT1*) was found to be the target gene of lncRNA MSTRG.56765.1 in this study (Table 2). Based on all above evidences and results, it is proposed that lncRNAs may play important roles in flag leaf senescence by involving in hormone pathways (Fig. 12a).

### Involvement of lncRNAs in flag leaf senescence by targeting genes related to redox metabolism

The redox state of cells is important for plant. Reactive oxygen species (ROS), which are continually produced by aerobic metabolism, are thought as cytotoxic molecules when in high levels, while low levels of ROS can act as signaling molecules to regulate plant development, stress responses, programmed cell death, and senescence (Jajic et al. 2015). In present study, interested senescence-associated lncRNAs were found to target a large number of mRNAs enriched in ROS-related GO terms, such as ‘oxidation–reduction process (GO:0055114)’, ‘response to oxygen-containing compound (GO:1901700)’ and ‘cellular response to oxygen-containing compound (GO:1901701)’ (Fig. 7, Table S11). Ascorbate peroxidase (APX) enzyme plays an essential role in the control of intracellular H_2_O_2_ levels. And *OsAPX4* gene has been reported to play an important role in leaf senescence mediated by ROS signaling (Ribeiro et al. 2017). In this study, BGIOSGA026526 (*OsAPX4*) was predicted to be the target gene of lncRNA MSTRG.79278.1 (Table 2). NAD plays critical roles in cellular redox reactions and remains at a sufficient level in the cell to prevent cell death. *OsNaPRT1*, encoding the nicotinate phosphoribosyltransferase in the NAD salvage pathway, protects rice plant from premature leaf senescence by maintaining the redox balance (Wu et al. 2016). BGIOSGA013898 (*OsNaPRT1*) was predicted to be the target gene of lncRNA MSTRG.44302.1 (Table 2). It is suggested that senescence associated lncRNAs perform functions probably by regulating genes involved in the redox metabolism (Fig. 12a).

### Involvement of lncRNAs in flag leaf senescence by targeting genes for the degradation of chlorophyll and chloroplast

Leaf senescence is characterized by the gradual loss of green coloration, mainly due to chlorophyll degradation. Chlorophyll catabolic enzymes are needed to perform catalytic reactions during the chlorophyll degradation process, such as chlorophyll b reductases encoded by *NYC1* (Kusaba et al. 2007) and *NOL* (Sato et al. 2009), 7-hydroxymethyl chlorophyll a reductase encoded by *OsHCAR* (Piao et al. 2017), pheophorbide a oxygenase encoded by *OsPAO* (Tang et al. 2011), and red chlorophyll catabolite reductase encoded by *OsRCCR1* (Tang et al. 2011). In this study, BGIOSGA003046 (*NYC1*), BGIOSGA010125 (*NOL*), BGIOSGA010917 (*OsHCAR*), BGIOSGA011859 (*OsPAO*) and BGIOSGA032012 (*OsRCCR1*) were predicted to be the target gene of 21, 18, 3, 11 and 12 lncRNAs, respectively (Table 2). In addition, a lot of genes were found to play roles in regulation of chlorophyll degradation during senescence, such as *NYC3* (Morita et al. 2009), *NYC4* (Yamatani et al. 2013), and *SGRL* (Rong et al. 2013). And BGIOSGA022884 (*NYC3*), BGIOSGA024095 (*NYC4*) and BGIOSGA029383 (*SGRL*) were predicted to be the target gene of 7, 1 and 17 lncRNAs, respectively (Table 2). *OsAkαGal* is reported to degrade thylakoid membranes in the chloroplast during leaf senescence in rice (Lee et al. 2009). In this study, BGIOSGA028915 (*OsAkαGal*) was predicted to be the target gene of lncRNA MSTRG.54054.1 (Table 2). In addition, GO analysis revealed that senescence associated lncRNAs targeted mRNAs involving in ‘chlorophyll metabolic process (GO:0015994)’, ‘chloroplast stroma (GO:0009570)’, ‘chloroplast envelope (GO:0009941)’ and ‘plasma membrane (GO:0005886)’ (Table S11). And KEGG analysis found that senescence associated lncRNAs targeted mRNAs involving in ‘photosynthesis-antenna proteins (ko00196) (Fig. 8a). To summarize, it is apparent that lncRNAs may participate in flag leaf senescence by regulation the degradation process of chlorophyll and chloroplast (Fig. 12a).

### Involvement of lncRNAs in flag leaf senescence by targeting lesion mimic genes

Early leaf senescence is usually present as one of the characteristics for lesion mimic mutants. Functional analysis of the genes responsible for lesion mimic mutants have provided a unique tool for dissecting possible molecular mechanism and regulatory pathway during leaf senescence. The rice lesion-mimic gene *SPL4* encodes a plant spastin that inhibits ROS accumulation in leaf development and functions in leaf senescence, and its mutant showed low seed setting rate and panicle number, but increased 500-grain weight (Song et al. 2019). *SPL28* appears to be involved in the regulation of vesicular trafficking, and SPL28 dysfunction causes the formation of hypersensitive response-like lesions, leading to the initiation of leaf senescence, small panicles, produced wizened grains, and few viable seeds (Qiao et al. 2010). *SPL29* encodes UDP-*N*-acetylglucosamine pyrophosphorylase 1, and its functional inactivation induces early leaf senescence and defense responses in rice, accompanying with decreased panicle length, grain number, and thousand-grain weight (Wang et al. 2015). The *OsPLS1* gene encoding vacuolar H^+^-ATPase subunit A1 (VHAA1), is implicated in leaf senescence through a combination of ROS and SA signals (Yang et al. 2016). In present study, these lesion mimic genes BGIOSGA022234 (*SPL4*), BGIOSGA004283 (*SPL28*), BGIOSGA028186 (*SPL29*), and BGIOSGA020739 (*OsPLS1*) were predicted to be the target gene of one or two lncRNAs (Table 2). It is proposed to be an important way that lncRNAs participates in leaf senescence though regulating lesion mimic genes (Fig. 12a).

### Involvement of lncRNAs in flag leaf senescence by targeting genes in other biological processes

Calcium-dependent protein kinases as a type of calcium sensor, perform multiple biological function in plants including senescence and cell death. *OsCPK12* encodes a calcium-dependent protein kinase. The mutant of *OsCPK12* triggers the premature leaf senescence, while the overexpression of *OsCPK12* may delay its growth period and provide the potentially positive effect on productivity in rice (Wang et al. 2019b). In our study, BGIOSGA014554 (*OsCPK12*) is predicted to be the target gene of lncRNA MSTRG.51442.2 (Table 2). Protein glycosylation may paly critical roles in flag leaf senescence (Huang et al. 2019a). Glycosyltransferases catalyzing the transfer of sugar moieties for glycosylation. *PLS2*, a putative glycosyltransferase-encoding gene, is involved in leaf senescence (Wang et al. 2018b). In present investigation, BGIOSGA012281 (*PLS2*) is identified to be the target gene of lncRNA MSTRG.20126.1 (Table 2). Senescence could be induced by abiotic stresses. A salt-induced protein gene *salT* was found to affect the leaf senescence induced by natural and dark conditions (Zhu et al. 2019), and BGIOSGA001700 (*salT*) was predicted to be the target gene of 10 lncRNAs (Table 2). *OsGDCH* encodes the H-protein subunit of the glycine decarboxylase complex, playing a major role in photorespiration, and knockdown of *GDCH* gene reveals reactive oxygen species-induced leaf senescence in rice (Zhou et al. 2013). BGIOSGA033278 (*OsGDCH*) identified in this study was predicted to be the target gene of three lncRNAs MSTRG.44376.1, MSTRG.24808.2 and MSTRG.65878.1 (Table 2). Transgenic rice overexpressing an F-box protein containing a Kelch repeat motif OsFBK12 delayed in leaf senescence and increased seed size, while knockdown lines could promote the senescence program (Chen et al. 2013). BGIOSGA011953 (*OsFBK12*) identified in this study was predicted to be the target gene of 15 lncRNAs (Table 2). It is seemed that lncRNA can be involved in flag leaf senescence by participating in several different biological processes (Fig. 12a).

### Vital roles of key lncRNAs in lncRNAs-miRNAs-mRNAs network

It has been reported that lncRNAs can competitively bind to miRNAs through miRNA response elements and affect the expression of genes (Salmena et al. 2011). In the present study, based on the interested down-regulated and up-regulated lncRNAs, and their co-expressed mRNAs, two ceRNA networks were constructed, from which, some key lncRNAs may regulate a large number of mRNAs through miRNAs to participate in flag leaf senescence (Fig. 10).

The roles of miR164 and its NAC target genes were reported to involve in regulating a numerous of biological processes. It has been reported that miR164 is a conserved microRNA with miR164-targeted *NAC* genes negatively regulating drought resistance in rice (Fang et al. 2014). In Kiwifruit, a genome-wide analysis of coding and non-coding RNA revealed a conserved miR164-*NAC* regulatory pathway playing key roles in fruit ripening (Wang et al. 2019c). A conserved role for the NAM/miR164 developmental module revealed a common mechanism underlying carpel margin fusion in Monocarpous and Syncarpous Eurosids (Vialette-Guiraud et al. 2016). In addition, the rice transcription factor *ONAC011* could promote leaf senescence and accelerate heading time in rice (El Mannai et al. 2017). The *OsNAC2* was found to promote leaf senescence via abscisic acid synthesis (Mao et al. 2017). In ceRNA network constructed by up-regulated lncRNAs and their co-expressed mRNAs, lncRNA MSTRG.62092.1 was predicted to regulate four NAC TFs, BGIOSGA023457 (*ONAC011*), BGIOSGA016546 (*OsNAC2*), BGIOSGA037778, BGIOSGA008492, acting as the target genes of osa-miR164a and osa-miR164e (Fig. 10f). These indicated that a conserved miR164-*NAC* regulatory pathway may be presented in the process of flag leaf senescence. Phytohormones including jasmonates and abscisic acid, were reported to play key roles in leaf senescence. Epigenetically regulation of*OsPME1* was reported to affect the content of jasmonates and thus play an important role in regulating leaf senescence (Fang et al. 2016). In abscisic acid biosynthetic pathway, 9-cis-epoxycarotenoid dioxygenase (NCED) is the key rate-limiting enzyme. The CRISPR/Cas9 knockout of abscisic acid-biosynthesis genes *OsNCED3* was identified to delay leaf senescence in transgenic rice, while its overexpression of *OsNCED3* could promote leaf senescence (Huang et al. 2018). And *OsNCED5* overexpression increased abscisic acid level, enhanced tolerance to the stresses and accelerated leaf senescence (Huang et al. 2019b). Moreover, the TF *ONAC066* can function as a positive regulator to resist drought induced plant death (Yuan et al. 2019). In this study, mRNAs BGIOSGA004591, BGIOSGA025169, and BGIOSGA013656 were identified as *OsPME1*, an abscisic acid-biosynthesis gene *OsNCED4*, and *ONAC066*, respectively. And lncRNAs MSTRG.31014.21, MSTRG.31014.36 and MSTRG.39310.1 were proposed to regulate these three well-studied SAGs and another ten unreported TFs through osa-miR5809 (Fig. 10g).

Chlorophyll degradation happens during leaf senescence. *NYC1* is one of the key enzymes in the chlorophyll degradation pathway (Kusaba et al. 2007). And BGIOSGA003046 (*NYC1*) was identified in our ceRNA networks, probably regulated by lncRNAs MSTRG.80924.2 and MSTRG.27317.2 through binding with osa-miR156k (Fig. 10h).

Taken together, these ceRNA network results provided several key lncRNAs involving in flag leaf senescence (Fig. 12b), and might lay a foundation for investigating the important function of candidate lncRNAs in flag leaf senescence through transgenic technology.

## Conclusion

Overall, in this study, a total of 3953 lncRNAs and 38757 mRNAs were identified in flag leaves of rice at five developmental stages from normal to senescence by using the genome-wide high throughput sequencing. And 343 lncRNAs and 9412 mRNAs were differentially expressed in ten pairwise comparison groups. LncRNAs showing continuous negative/positive correlation with flag leaf senescence were additionally identified by WGCNA. GO enrichment results suggested that the senescence negatively and positively associated DE lncRNAs targeted their co-expressed DE mRNAs involving in many biological processes including transcription, hormone response, oxidation–reduction process and substance metabolism.

Furthermore, a great number of lncRNAs regulating transcription factors and well-studied SAGs were identified, providing the evidences for the credibility of the results in this study. One lncRNA was found to possibly participate in multiple senescence-associated biological processes. And lncRNAs like MSTRG.84624.1, MSTRG.76387.1, MSTRG.8133.1, MSTRG.77321.3, MSTRG.79278.1, MSTRG.552.1, MSTRG.65878.1, MSTRG.4640.1, MSTRG.4077.5, MSTRG.19268.3, MSTRG.29832.1 and MSTRG.12677.1, which participated in more than four different biological processes, were recommended to be prior candidates for transgenic analysis (Fig. 12a, Table S15). Finally, the ceRNA networks of the senescence-associated lncRNAs regulating the expression of mRNAs through miRNAs were also analyzed and partially confirmed by expression profile analysis. And lncRNAs like MSTRG.31014.21, MSTRG.31014.36 and MSTRG.39310.1, which probably regulated multiple senescence-related mRNAs, were recommended to be prior candidates for transgenic analysis (Figs. 10, 12b). The present study provided novel insight into regulatory functions of lncRNAs, and laid a foundation for functional studies on mediating leaf senescence by candidate lncRNAs in rice.

## Supplementary Information

Below is the link to the electronic supplementary material.

Supplementary file1 (XLSX 4679 KB)
Table S1 List of primers for quantitative real-time PCR.

Supplementary file2 (XLSX 179 KB)
Table S2 The identified 38757 mRNAs during flag leaf senescence in rice.

Supplementary file3 (XLSX 93 KB)
Table S3 The identified 3953 lncRNAs during flag leaf senescence in rice.

Supplementary file4 (XLSX 52 KB)
Table S4 The conservation analysis of the identified 3953 lncRNAs among 10 plant species.

Supplementary file5 (XLSX 1232 KB)
Table S5 The differentially expressed lncRNAs in 10 comparison groups during flag leaf senescence in rice.

Supplementary file6 (XLSX 10678 KB)
Table S6 The differentially expressed mRNAs in 10 comparison groups during flag leaf senescence in rice.

Supplementary file7 (XLSX 95 KB)
Table S7 The predicted *cis*- and *trans*-target genes of 3953 lncRNAs.

Supplementary file8 (XLSX 395 KB)
The 22 down-regulated lncRNAs and their co-expressed 812 differentially expressed mRNAs in ‘ME black’ module, and 48 up-regulated lncRNAs and their co-expressed 1209 differentially expressed mRNAs in ‘ME blue’ module.

Supplementary file9 (XLSX 162 KB)
Table S9 The target genes of 22 down-regulated differentially expressed lncRNAs in ‘ME black’ module and 48 up-regulated differentially expressed lncRNAs in ‘ME blue’ module.

Supplementary file10 (XLSX 102 KB)
Table S10 The 378 differentially expressed target mRNAs regulated by 22 differentially expressed lncRNAs in 'ME black' module and 941 differentially expressed target mRNAs regulated by 48 differentially expressed lncRNAs in 'ME blue' module.

Supplementary file11 (XLSX 12 KB)
Table S11 Gene Ontology enrichment analysis on 378 differentially expressed target mRNAs regulated by 22 differentially expressed lncRNAs in 'ME black' module and 941 differentially expressed target mRNAs regulated by 48 differentially expressed lncRNAs in 'ME blue' module.

Supplementary file12 (XLSX 45 KB)
Table S12 KEGG enrichment analysis on 378 differentially expressed target mRNAs regulated by 22 differentially expressed lncRNAs in 'ME black' module and 941 differentially expressed target mRNAs regulated by 48 differentially expressed lncRNAs in 'ME blue' module.

Supplementary file13 (XLSX 23 KB)
Table S13 The transcription factors regulated by 22 and 48 differentially expressed lncRNAs in 'ME black' and ‘ME blue’ module.

Supplementary file14 (DOCX 19 KB)
Table S14 The details of multiple well-studied senescence-associated genes regulated by one lncRNA.

Supplementary file15 (XLSX 16 KB)
Table S15 The summary on the possible involvement of one lncRNA in multiple senescence-associated biological processes by targeting well-studied senescence-associated genes.

## Data Availability

All data generated or analyzed during this study are included in this published article and its supplementary information files.

## Abbreviations

ceRNA: Competing endogenous RNA
CNCI: Coding-non-coding index
CPAT: Coding potential assessing tool
CPC: Coding potential calculator
DE: Differentially expressed
GO: Gene ontology
KEGG: Kyoto encyclopedia of genes and genomes
lncRNA: Long non-coding RNA
MiRNA: MicroRNA
Pfam: Protein folding domain database
qRT-PCR: Quantitative real-time polymerase chain reaction
ROS: Reactive oxygen species
TFs: Transcription factors
WGCNA: Weighted gene co-expression network analysis

## References

[CR1] Arnao MB, Hernández-Ruiz J (2018). Melatonin and its relationship to plant hormones. Ann Bot.

[CR2] Cabili MN, Trapnell C, Goff L, Koziol M, Tazon-Vega B, Regev A, Rinn JL (2011). Integrative annotation of human large intergenic noncoding RNAs reveals global properties and specific subclasses. Genes Dev.

[CR3] Cesana M, Cacchiarelli D, Legnini I, Santini T, Sthandier O, Chinappi M, Tramontano A, Bozzoni I (2011). A long noncoding RNA controls muscle differentiation by functioning as a competing endogenous RNA. Cell.

[CR4] Chen Y, Xu Y, Luo W, Li W, Chen N, Zhang D, Chong K (2013). The F-Box protein OsFBK12 targets OsSAMS1 for degradation and affects pleiotropic phenotypes, including leaf senescence in rice. Plant Physiol.

[CR5] Chen X, Ma C, Chen C, Lu Q, Shi W, Liu Z, Wang H, Guo H (2017). Integration of lncRNA–miRNA–mRNA reveals novel insights into oviposition regulation in honey bees. Peer J.

[CR6] Conesa A, Gotz S, Garcia-Gomez JM, Terol J, Talon M, Robles M (2005). Blast2GO: a universal tool for annotation, visualization and analysis in functional genomics research. Bioinformatics.

[CR7] Cui J, Luan Y, Jiang N, Bao H, Meng J (2017). Comparative transcriptome analysis between resistant and susceptible tomato allows the identification of lncRNA16397 conferring resistance to *Phytophthora infestans* by co-expressing glutaredoxin. Plant J.

[CR8] Dai X, Zhuang Z, Zhao PX (2018). psRNATarget: a plant small RNA target analysis server (2017 release). Nucleic Acids Res.

[CR9] Deng F, Zhang X, Wang W, Yuan R, Shen F (2018). Identification of *gossypium hirsutum* long non-coding RNAs (lncRNAs) under salt stress. BMC Plant Biol.

[CR10] Ding Z, Tie W, Fu L, Yan Y, Liu G, Yan W, Li Y, Wu C, Zhang J, Hu W (2019). Strand-specific RNA-seq based identification and functional prediction of drought-responsive lncRNAs in cassava. BMC Genomics.

[CR11] El Mannai Y, Akabane K, Hiratsu K, Satoh-Nagasawa N, Wabiko H (2017). The NAC transcription factor gene OsY37 (ONAC011) promotes leaf senescence and accelerates heading time in rice. Int J Mol Sci.

[CR12] Fabbri M, Calin GA (2010). Beyond genomics: interpreting the 93% of the human genome that does not encode proteins. Curr Opin Drug Discov Dev.

[CR13] Fang Y, Xie K, Xiong L (2014). Conserved miR164-targeted NAC genes negatively regulate drought resistance in rice. J Exp Bot.

[CR14] Fang C, Zhang H, Wan J (2016). Control of leaf senescence by an MeOH-Jasmonates cascade that is epigenetically regulated by OsSRT1 in rice. Mol Plant.

[CR15] Fang J, Zhang F, Wang H, Wang W, Zhao F, Li Z, Sun C, Chen F, Xu F, Chang S, Wu L, Bu Q, Wang P, Xie J, Chen F, Huang X, Zhang Y, Zhu X, Han B, Deng X, Chu C (2019). Ef-cd locus shortens rice maturity duration without yield penalty. Proc Natl Acad Sci USA.

[CR16] Finn RD, Bateman A, Clements J, Coggill P, Eberhardt RY, Eddy SR, Heger A, Hetherington K, Holm L, Mistry J, Sonnhammer ELL, Tate J, Punta M (2013). Pfam: the protein families database. Nucleic Acids Res.

[CR17] Franco-Zorrilla JM, Valli A, Todesco M, Mateos I, Puga MI, Rubio-Somoza I, Leyva A, Weigel D, García JA, Paz-Ares J (2007). Target mimicry provides a new mechanism for regulation of microRNA activity. Nat Genet.

[CR18] He X, Guo S, Wang Y, Wang L, Shu S, Sun J (2019). Systematic identification and analysis of heat-stress-responsive lncRNAs, circRNAs and miRNAs with associated co-expression and ceRNA networks in cucumber (*Cucumis sativus* L.). Physiol Plant.

[CR19] Huang Y, Guo Y, Liu Y, Zhang F, Wang Z, Wang H, Wang F, Li D, Mao D, Luan S, Liang M, Chen L (2018). 9-cis-epoxycarotenoid dioxygenase 3 regulates plant growth and enhances multi-abiotic stress tolerance in rice. Front Plant Sci.

[CR20] Huang X, Zhang H, Liao J, Wei L, Guo R, Xiao W, Kuang W, Huang Y, Wang Z (2019). Qualitative analysis of N-linked glycoproteome in senescent flag leaf of rice. Plant Growth Regul.

[CR21] Huang Y, Jiao Y, Xie N, Guo Y, Zhang F, Xiang Z, Wang R, Wang F, Gao Q, Tian L, Li D, Chen L, Liang M (2019). OsNCED5, a 9-cis-epoxycarotenoid dioxygenase gene, regulates salt and water stress tolerance and leaf senescence in rice. Plant Sci.

[CR22] Jabnoune M, Secco D, Lecampion C, Robaglia C, Shu Q, Poirier Y (2013). A rice *cis*-natural antisense RNA acts as a translational enhancer for its cognate mRNA and contributes to phosphate homeostasis and plant fitness. Plant Cell.

[CR23] Jajic I, Sarna T, Strzalka K (2015). Senescence, stress, and reactive oxygen species. Plants.

[CR24] Jiang N, Cui J, Shi Y, Yang G, Zhou X, Hou X, Meng J, Luan Y (2019). Tomato lncRNA23468 functions as a competing endogenous RNA to modulate NBS-LRR genes by decoying miR482b in the tomato-*Phytophthora infestans* interaction. Hortic Res.

[CR25] Kim D, Langmead B, Salzberg SL (2015). HISAT: a fast spliced aligner with low memory requirements. Nat Methods.

[CR26] Kim J, Woo HR, Nam HG (2016). Toward systems understanding of leaf senescence: an integrated multi-omics perspective on leaf senescence research. Mol Plant.

[CR27] Kim HJ, Ryu H, Hong SH, Woo HR, Lim PO, Lee IC, Sheen J, Nam HG, Hwang I (2006). Cytokinin-mediated control of leaf longevity by AHK3 through phosphorylation of ARR2 in Arabidopsis. Proc Natl Acad Sci USA.

[CR28] Kong Z, Li M, Yang W, Xu W, Xue Y (2006). A novel nuclear-localized CCCH-type zinc finger protein, OsDOS, is involved in delaying leaf senescence in rice. Plant Physiol.

[CR29] Kong L, Zhang Y, Ye Z, Liu X, Zhao S, Wei L, Gao G (2007). CPC: assess the protein-coding potential of transcripts using sequence features and support vector machine. Nucleic Acids Res.

[CR30] Kudo T, Makita N, Kojima M, Tokunaga H, Sakakibara H (2012). Cytokinin activity of cis-zeatin and phenotypic alterations induced by overexpression of putative cis-zeatin-O-glucosyltransferase in rice. Plant Physiol.

[CR31] Kusaba M, Ito H, Morita R, Iida S, Sato Y, Fujimoto M, Kawasaki S, Tanaka R, Hirochika H, Nishimura M, Tanaka A (2007). Rice NON-YELLOW COLORING1 is involved in light-harvesting complex II and grana degradation during leaf senescence. Plant Cell.

[CR32] Langfelder P, Horvath S (2008). WGCNA: an R package for weighted correlation network analysis. BMC Bioinform.

[CR33] Lee K, Back K (2017). Overexpression of rice serotonin N-acetyltransferase 1 in transgenic rice plants confers resistance to cadmium and senescence and increases grain yield. J Pineal Res.

[CR34] Lee RH, Hsu JH, Huang HJ, Lo SF, Grace-Chen SC (2009). Alkaline a-galactosidase degrades thylakoid membranes in the chloroplast during leaf senescence in rice. New Phytol.

[CR35] Lee SH, Sakuraba Y, Lee T, Kim KW, An G, Lee HY, Paek NC (2015). Mutation of *Oryza sativa* CORONATINE INSENSITIVE 1b (OsCOI1b) delays leaf senescence. J Integr Plant Biol.

[CR36] Leng Y, Ye G, Zeng D (2017). Genetic dissection of leaf senescence in rice. Int J Mol Sci.

[CR37] Liang C, Zheng G, Li W, Wang Y, Hu B, Wang H, Wu H, Qian Y, Zhu X, Tan D, Chen S, Chu C (2015). Melatonin delays leaf senescence and enhances salt stress tolerance in rice. J Pineal Res.

[CR38] Lichtenthaler HK (1987). Chlorophylls and carotenoids: pigments of photosynthetic biomembranes. Methods Enzymol.

[CR39] Lim PO, Kim HJ, Nam HG (2007). Leaf senescence. Annu Rev Plant Biol.

[CR40] Liu J, Wang H, Chua N (2015). Long noncoding RNA transcriptome of plants. Plant Biotechnol J.

[CR41] Liu X, Hao L, Li D, Zhu L, Hu S (2015). Long non-coding RNAs and their biological roles in plants. Genomics Proteomics Bioinform.

[CR42] Liu X, Li D, Zhang D, Yin D, Zhao Y, Ji C, Zhao X, Li X, He Q, Chen R, Hu S, Zhu L (2018). A novel antisense long noncoding RNA, TWISTED LEAF, maintains leaf blade flattening by regulating its associated sense R2R3-MYB gene in rice. New Phytol.

[CR43] Liu F, Xu Y, Chang K, Li S, Liu Z, Qi S, Jia J, Zhang M, Crawford NM, Wang Y (2019). The long noncoding RNA T5120 regulates nitrate response and assimilation in Arabidopsis. New Phytol.

[CR44] Mao C, Lu S, Lv B, Zhang B, Shen J, He J, Luo L, Xi D, Chen X, Ming F (2017). A rice NAC transcription factor promotes leaf senescence via ABA biosynthesis. Plant Physiol.

[CR45] Mattick JS, Rinn JL (2015). Discovery and annotation of long noncoding RNAs. Nat Struct Mol Biol.

[CR46] Morita R, Sato Y, Masuda Y, Nishimura M, Kusaba M (2009). Defect in non-yellow coloring 3, an α/β hydrolase-fold family protein, causes a stay-green phenotype during leaf senescence in rice. Plant J.

[CR47] Necsulea A, Soumillon M, Warnefors M, Liechti A, Daish T, Zeller U, Baker JC, Grutzner F, Kaessmann H (2014). The evolution of lncRNA repertoires and expression patterns in tetrapods. Nature.

[CR48] Pertea M, Kim D, Pertea GM, Leek JT, Salzberg SL (2016). Transcript-level expression analysis of RNA-seq experiments with HISAT, StringTie and Ballgown. Nat Protoc.

[CR49] Piao W, Han SH, Sakuraba Y, Paek NC (2017). Rice 7-hydroxymethyl chlorophyll a reductase is involved in the promotion of chlorophyll degradation and modulates cell death signaling. Mol Cells.

[CR50] Podzimska-Sroka D, O'Shea C, Gregersen P, Skriver K (2015). NAC transcription factors in senescence: from molecular structure to function in crops. Plants.

[CR51] Qiao Y, Jiang W, Lee JH, Park BS, Choi MS (2010). SPL28 encodes a clathrin-associated adaptor protein complex 1, medium subunit l1 (AP1M1) and is responsible for spotted leaf and early senescence in rice (*Oryza sativa*). New Phytol.

[CR52] Ren G, Fan X, Liu T, Wang S, Zhao G (2018). Genome-wide analysis of differentially expressed profiles of mRNAs, lncRNAs and circRNAs during *Cryptosporidium baileyi* infection. BMC Genomics.

[CR53] Ribeiro CW, Korbes AP, Garighan JA (2017). Rice peroxisomal ascorbate peroxidase knockdown affects ROS signaling and triggers early leaf senescence. Plant Sci.

[CR54] Rinn JL, Kertesz M, Wang JK, Squazzo SL, Xu X, Brugmann SA, Goodnough LH, Helms JA, Farnham PJ, Segal E, Chang HY (2007). Functional demarcation of active and silent chromatin domains in human HOX loci by noncoding RNAs. Cell.

[CR55] Rong H, Tang Y, Zhang H, Wu P, Chen Y, Li M, Wu G, Jiang H (2013). The stay-green rice like (SGRL) gene regulates chlorophyll degradation in rice. J Plant Physiol.

[CR56] Salmena L, Poliseno L, Tay Y, Kats L, Pandolfi PP (2011). A ceRNA hypothesis: the Rosetta Stone of a hidden RNA language?. Cell.

[CR57] Sato Y, Morita R, Katsuma S, Nishimura M, Tanaka A, Kusaba M (2009). Two short-chain dehydrogenase/reductases, NON-YELLOW COLORING 1 and NYC1-LIKE, are required for chlorophyll b and light-harvesting complex II degradation during senescence in rice. Plant J.

[CR58] Sleutels F, Zwart R, Barlow DP (2002). The non-coding Air RNA is required for silencing autosomal imprinted genes. Nature.

[CR59] Song G, Kwon C, Kim S, Shim Y, Lim C, Koh H, An G, Kang K, Paek N (2019). The rice spotted leaf 4 (SPL4) encodes a plant spastin that inhibits ROS accumulation in leaf development and functions in leaf senescence. Front Plant Sci.

[CR60] Stadler PF (2014). Class-specific prediction of ncRNAs. Methods Mol Biol.

[CR61] Strawn MA, Marr SK, Inoue K, Inada N, Zubieta C, Wildermuth MC (2007). Arabidopsis isochorismate synthase functional in pathogen-induced salicylate biosynthesis exhibits properties consistent with a role in diverse stress responses. J Biol Chem.

[CR62] Sun L, Luo H, Bu D, Zhao G, Yu K, Zhang C, Liu Y, Chen R, Zhao Y (2013). Utilizing sequence intrinsic composition to classify protein-coding and long non-coding transcripts. Nucleic Acids Res.

[CR63] Tang Y, Li M, Chen Y, Wu P, Wu G, Jiang H (2011). Knockdown of OsPAO and OsRCCR1 cause different plant death phenotypes in rice. J Plant Physiol.

[CR64] Tian Y, Bai S, Dang Z, Hao J, Zhang J, Hasi A (2019). Genome-wide identification and characterization of long non-coding RNAs involved in fruit ripening and the climacteric in *Cucumis melo*. BMC Plant Biol.

[CR65] Vialette-Guiraud ACM, Chauvet A, Gutierrez-Mazariegos J, Eschstruth A, Ratet P, Scutt CP (2016). A conserved role for the NAM/miR164 developmental module reveals a common mechanism underlying carpel margin fusion in Monocarpous and Syncarpous Eurosids. Front Plant Sci.

[CR66] Wang L, Park HJ, Dasari S, Wang S, Kocher J, Li W (2013). CPAT: coding-potential assessment tool using an alignment-free logistic regression model. Nucleic Acids Res.

[CR67] Wang Z, Wang Y, Hong X, Hu D, Liu C, Yang J, Li Y, Huang Y, Feng Y, Gong H, Li Y, Fang G, Tang H, Li Y (2015). Functional inactivation of UDP-N-acetylglucosamine pyrophosphorylase 1 (UAP1) induces early leaf senescence and defence responses in rice. J Exp Bot.

[CR68] Wang Z, Wang Y, Yang J, Hu K, An B, Deng X, Li Y (2016). Reliable selection and holistic stability evaluation of reference genes for rice under 22 different experimental conditions. Appl Biochem Biotechnol.

[CR69] Wang M, Wu H, Fang J, Chu C, Wang X (2017). A long noncoding RNA involved in rice reproductive development by negatively regulating osa-miR160. Sci Bull.

[CR70] Wang M, Zhang T, Peng H, Luo S, Tan J, Jiang K, Heng Y, Zhang X, Guo X, Zheng J, Cheng Z (2018). Rice premature leaf senescence 2, encoding a glycosyltransferase (GT), is involved in leaf senescence. Front Plant Sci.

[CR71] Wang Y, Luo X, Sun F, Hu J, Zha X, Su W, Yang J (2018). Overexpressing lncRNA LAIR increases grain yield and regulates neighbouring gene cluster expression in rice. Nat Commun.

[CR72] Wang W, Wang J, Wu Y, Li D, Allan AC, Yin X (2019). Genome-wide analysis of coding and non-coding RNA reveals a conserved miR164-NAC regulatory pathway for fruit ripening. New Phytol.

[CR73] Wang B, Zhang Y, Bi Z, Liu Q, Xu T, Yu N, Cao Y, Zhu A, Wu W, Zhan X, Anis GB, Yu P, Chen D, Cheng S, Cao L (2019). Impaired function of the calcium-dependent protein kinase, OsCPK12, leads to early senescence in rice (*Oryza sativa* L.). Front Plant Sci.

[CR74] Wang A, Hu J, Gao C, Chen G, Wang B, Lin C, Song L, Ding Y, Zhou G (2019). Genome-wide analysis of long non-coding RNAs unveils the regulatory roles in the heat tolerance of Chinese cabbage (*Brassica rapa* ssp.chinensis). Sci Rep.

[CR75] Wasternack C, Song S (2016). Jasmonates: biosynthesis, metabolism, and signaling by proteins activating and repressing transcription. J Exp Bot.

[CR76] Wu L, Ren D, Hu S, Li G, Dong G, Jiang L, Hu X, Ye W, Cui Y, Zhu L, Hu J, Zhang G, Gao Z, Zeng D, Qian Q, Guo L (2016). Down-regulation of a nicotinate phosphoribosyltransferase gene, OsNaPRT1, leads to withered leaf tips. Plant Physiol.

[CR77] Wu X, Shi T, Iqbal S, Zhang Y, Liu L, Gao Z (2019). Genome-wide discovery and characterization of flower development related long non-coding RNAs in *Prunus mume*. BMC Plant Biol.

[CR78] Yamatani H, Sato Y, Masuda Y, Kato Y, Morita R, Fukunaga K, Nagamura Y, Nishimura M, Sakamoto W, Tanaka A, Kusaba M (2013). NYC4, the rice ortholog of Arabidopsis THF1, is involved in the degradation of chlorophyll—protein complexes during leaf senescence. Plant J.

[CR79] Yang X, Gong P, Li K, Huang F, Cheng F, Pan G (2016). A single cytosine deletion in the OsPLS1 gene encoding vacuolar-type H+-ATPase subunit A1 leads to premature leaf senescence and seed dormancy in rice. J Exp Bot.

[CR80] Yu Y, Zhou YF, Feng YZ, He H, Lian JP, Yang YW, Lei MQ, Zhang YC, Chen YQ (2020). Transcriptional landscape of pathogen-responsive lncRNAs in rice unveils the role of ALEX1 in jasmonate pathway and disease resistance. Plant Biotechnol J.

[CR81] Yuan X, Wang H, Cai J, Bi Y, Li D, Song F (2019). Rice NAC transcription factor *ONAC066* functions as a positive regulator of drought and oxidative stress response. BMC Plant Biol.

[CR82] Zhang W, Zhou X, Wen C (2012). Modulation of ethylene responses by OsRTH1 overexpression reveals the biological significance of ethylene in rice seedling growth and development. J Exp Bot.

[CR83] Zhao J, Sun BK, Erwin JA, Song JJ, Lee JT (2008). Polycomb proteins targeted by a short repeat RNA to the mouse X chromosome. Science.

[CR84] Zhou Q, Yu Q, Wang Z, Pan Y, Lv W, Zhu L, Chen R, He G (2013). Knockdown of GDCH gene reveals reactive oxygen species-induced leaf senescence in rice. Plant Cell Environ.

[CR85] Zhu Q, Wang M (2012). Molecular functions of long non-coding RNAs in plants. Genes.

[CR86] Zhu B, Yang Y, Li R, Fu D, Wen L, Luo Y, Zhu H (2015). RNA sequencing and functional analysis implicate the regulatory role of long non-coding RNAs in tomato fruit ripening. J Exp Bot.

[CR87] Zhu K, Tao H, Xu S, Li K, Zafar S, Cao W, Yang Y (2019). Overexpression of salt-induced protein (salT) delays leaf senescence in rice. Genet Mol Biol.

[CR88] Zuo J, Wang Y, Zhu B, Luo Y, Wang Q, Gao L (2019). Network analysis of noncoding RNAs in pepper provides insights into fruit ripening control. Sci Rep.

