# Supplementary material for: Genome-wide identification and characterization of long non-coding RNAs involved in flag leaf senescence of rice
Source: Plant Mol Biol. 2021 Feb 11;105(6):655–84. doi: 10.1007/s11103-021-01121-3 (PMC7985109; doi:10.1007/s11103-021-01121-3)
Supplement: Supplementary file 14 — Supplementary file14 (DOCX 19 KB) Table S14 The details of multiple well-studied senescence-associated genes regulated by one lncRNA. [file 11103_2021_1121_MOESM14_ESM.docx]

**Table S15** The summary on the possible involvement of one lncRNA in multiple senescence-associated biological processes by targeting well-studied senescence-associated genes

| **lncRNA ID** | **Biological process** | **Target SAG ID** | **Gene name** |
| --- | --- | --- | --- |
| MSTRG.84624.1 | Transcription regulation | BGIOSGA023457 | *ONAC011* |
|  | Hormone pathways | BGIOSGA019953 | *OsCOI1b* |
|  | Degradation of chlorophyll and chloroplast | BGIOSGA003046 | *NYC1* |
|  |  | BGIOSGA011859 | *OsPAO* |
|  |  | BGIOSGA022884 | *NYC3* |
|  |  | BGIOSGA032012 | *OsRCCR1* |
|  | Lesion mimic genes | BGIOSGA020739 | *OsPLS1* |
|  |  | BGIOSGA022234 | *SPL4* |
|  | Others | BGIOSGA011953 | *OsFBK12* |
| MSTRG.76387.1 | Transcription regulation | BGIOSGA023457 | *ONAC011* |
|  | Hormone pathways | BGIOSGA019953 | *OsCOI1b* |
|  |  | BGIOSGA013214 | *OsNCED3* |
|  | Degradation of chlorophyll and chloroplast | BGIOSGA003046 | *NYC1* |
|  |  | BGIOSGA010125 | *NOL* |
|  |  | BGIOSGA011859 | *OsPAO* |
|  |  | BGIOSGA022884 | *NYC3* |
|  |  | BGIOSGA029383 | *SGRL* |
|  |  | BGIOSGA032012 | *OsRCCR1* |
|  |  | BGIOSGA010917 | *OsHCAR* |
|  | Lesion mimic genes | BGIOSGA022234 | *SPL4* |
|  | Others | BGIOSGA011953 | *OsFBK12* |
| MSTRG.8133.1 | Transcription regulation | BGIOSGA023457 | *ONAC011* |
|  | Hormone pathways | BGIOSGA004591 | *OsPME1* |
|  | Degradation of chlorophyll and chloroplast | BGIOSGA003046 | *NYC1* |
|  |  | BGIOSGA029383 | *SGRL* |
|  | Lesion mimic genes | BGIOSGA020739 | *OsPLS1* |
|  | Others | BGIOSGA011953 | *OsFBK12* |
| MSTRG.77321.3 | Transcription regulation | BGIOSGA023457 | *ONAC011* |
|  | Hormone pathways | BGIOSGA013214 | *OsNCED3* |
|  | Degradation of chlorophyll and chloroplast | BGIOSGA011859 | *OsPAO* |
|  |  | BGIOSGA003046 | *NYC1* |
|  |  | BGIOSGA010125 | *NOL* |
|  |  | BGIOSGA029383 | *SGRL* |
|  |  | BGIOSGA032012 | *OsRCCR1* |
|  | Others | BGIOSGA011953 | *OsFBK12* |
|  |  | BGIOSGA001700 | *salT* |
| MSTRG.79278.1 | Transcription regulation | BGIOSGA023457 | *ONAC011* |
|  | Hormone pathways | BGIOSGA019953 | *OsCOI1b* |
|  |  | BGIOSGA013214 | *OsNCED3* |
|  | Degradation of chlorophyll and chloroplast | BGIOSGA003046 | *NYC1* |
|  |  | BGIOSGA010125 | *NOL* |
|  |  | BGIOSGA011859 | *OsPAO* |
|  |  | BGIOSGA029383 | *SGRL* |
|  |  | BGIOSGA032012 | *OsRCCR1* |
|  | Redox metabolism | BGIOSGA026526 | *OsAPX4* |
| MSTRG.552.1 | Transcription regulation | BGIOSGA023457 | *ONAC011* |
|  | Hormone pathways | BGIOSGA019953 | *OsCOI1b* |
|  |  | BGIOSGA013214 | *OsNCED3* |
|  | Degradation of chlorophyll and chloroplast | BGIOSGA003046 | *NYC1* |
|  |  | BGIOSGA010125 | *NOL* |
|  |  | BGIOSGA011859 | *OsPAO* |
|  |  | BGIOSGA022884 | *NYC3* |
|  |  | BGIOSGA029383 | *SGRL* |
|  |  | BGIOSGA032012 | *OsRCCR1* |
|  |  | BGIOSGA010917 | *OsHCAR* |
|  | Others | BGIOSGA011953 | *OsFBK12* |
|  |  | BGIOSGA001700 | *salT* |
| MSTRG.65878.1 | Hormone pathways | BGIOSGA014571 | *cZOGT2* |
|  | Lesion mimic genes | BGIOSGA028186 | *SPL29* |
|  |  | BGIOSGA004283 | *SPL28* |
|  |  | BGIOSGA020739 | *OsPLS1* |
|  | Redox metabolism | BGIOSGA026526 | *OsAPX4* |
|  | Others | BGIOSGA033278 | *OsGDCH* |
| MSTRG.4640.1 | Transcription regulation | BGIOSGA023457 | *ONAC011* |
|  | Hormone pathways | BGIOSGA004316 | *OsRTH1* |
|  | Degradation of chlorophyll and chloroplast | BGIOSGA003046 | *NYC1* |
|  | Others | BGIOSGA011953 | *OsFBK12* |
|  |  | BGIOSGA001700 | *salT* |
| MSTRG.4077.5 | Transcription regulation | BGIOSGA023457 | *ONAC011* |
|  | Hormone pathways | BGIOSGA004316 | *OsRTH1* |
|  | Degradation of chlorophyll and chloroplast | BGIOSGA003046 | *NYC1* |
|  |  | BGIOSGA029383 | *SGRL* |
|  |  | BGIOSGA010125 | *NOL* |
|  | Others | BGIOSGA011953 | *OsFBK12* |
|  |  | BGIOSGA001700 | *salT* |
| MSTRG.19268.3 | Transcription regulation | BGIOSGA023457 | *ONAC011* |
|  | Hormone pathways | BGIOSGA013214 | *OsNCED3* |
|  |  | BGIOSGA019953 | *OsCOI1b* |
|  | Degradation of chlorophyll and chloroplast | BGIOSGA003046 | *NYC1* |
|  |  | BGIOSGA010125 | *NOL* |
|  |  | BGIOSGA011859 | *OsPAO* |
|  |  | BGIOSGA022884 | *NYC3* |
|  |  | BGIOSGA029383 | *SGRL* |
|  |  | BGIOSGA032012 | *OsRCCR1* |
|  |  | BGIOSGA010917 | *OsHCAR* |
|  | Others | BGIOSGA001700 | *salT* |
|  |  | BGIOSGA011953 | *OsFBK12* |
| MSTRG.29832.1 | Transcription regulation | BGIOSGA023457 | *ONAC011* |
|  | Hormone pathways | BGIOSGA013214 | *OsNCED3* |
|  | Degradation of chlorophyll and chloroplast | BGIOSGA003046 | *NYC1* |
|  |  | BGIOSGA010125 | *NOL* |
|  |  | BGIOSGA011859 | *OsPAO* |
|  |  | BGIOSGA029383 | *SGRL* |
|  |  | BGIOSGA032012 | *OsRCCR1* |
|  | Others | BGIOSGA001700 | *salT* |
|  |  | BGIOSGA011953 | *OsFBK12* |
| MSTRG.12677.1 | Transcription regulation | BGIOSGA023457 | *ONAC011* |
|  | Hormone pathways | BGIOSGA013214 | *OsNCED3* |
|  | Degradation of chlorophyll and chloroplast | BGIOSGA003046 | *NYC1* |
|  |  | BGIOSGA010125 | *NOL* |
|  |  | BGIOSGA011859 | *OsPAO* |
|  |  | BGIOSGA029383 | *SGRL* |
|  |  | BGIOSGA032012 | *OsRCCR1* |
|  | Others | BGIOSGA001700 | *salT* |
|  |  | BGIOSGA011953 | *OsFBK12* |
| MSTRG.8050.1 | Transcription regulation | BGIOSGA023457 | *ONAC011* |
|  | Degradation of chlorophyll and chloroplast | BGIOSGA003046 | *NYC1* |
|  |  | BGIOSGA010125 | *NOL* |
|  |  | BGIOSGA029383 | *SGRL* |
|  | Others | BGIOSGA001700 | *salT* |
|  |  | BGIOSGA011953 | *OsFBK12* |
| MSTRG.12677.3 | Transcription regulation | BGIOSGA023457 | *ONAC011* |
|  | Hormone pathways | BGIOSGA013214 | *OsNCED3* |
|  | Degradation of chlorophyll and chloroplast | BGIOSGA003046 | *NYC1* |
|  |  | BGIOSGA010125 | *NOL* |
|  |  | BGIOSGA011859 | *OsPAO* |
|  |  | BGIOSGA029383 | *SGRL* |
|  |  | BGIOSGA032012 | *OsRCCR1* |
|  |  | BGIOSGA024095 | *NYC4* |
| MSTRG.74673.4 | Transcription regulation | BGIOSGA023457 | *ONAC011* |
|  | Hormone pathways | BGIOSGA013214 | *OsNCED3* |
|  | Degradation of chlorophyll and chloroplast | BGIOSGA003046 | *NYC1* |
|  |  | BGIOSGA010125 | *NOL* |
|  |  | BGIOSGA029383 | *SGRL* |
| MSTRG.74673.3 | Transcription regulation | BGIOSGA023457 | *ONAC011* |
|  | Hormone pathways | BGIOSGA013214 | *OsNCED3* |
|  | Degradation of chlorophyll and chloroplast | BGIOSGA003046 | *NYC1* |
|  |  | BGIOSGA010125 | *NOL* |
|  |  | BGIOSGA029383 | *SGRL* |
| MSTRG.39310.1 | Degradation of chlorophyll and chloroplast | BGIOSGA011859 | *OsPAO* |
|  |  | BGIOSGA032012 | *OsRCCR1* |
|  |  | BGIOSGA022884 | *NYC3* |
|  | Lesion mimic genes | BGIOSGA020739 | *OsPLS1* |
|  |  | BGIOSGA022234 | *SPL4* |
|  | Others | BGIOSGA011953 | *OsFBK12* |
| MSTRG.42965.1 | Transcription regulation | BGIOSGA023457 | *ONAC011* |
|  | Degradation of chlorophyll and chloroplast | BGIOSGA003046 | *NYC1* |
|  |  | BGIOSGA010125 | *NOL* |
|  | Others | BGIOSGA011953 | *OsFBK12* |
|  |  | BGIOSGA001700 | *salT* |
| MSTRG.24808.2 | Hormone pathways | BGIOSGA014571 | *cZOGT2* |
|  | Lesion mimic genes | BGIOSGA004283 | *SPL28* |
|  |  | BGIOSGA028186 | *SPL29* |
|  | Others | BGIOSGA033278 | *OsGDCH* |
| MSTRG.30528.1 | Transcription regulation | BGIOSGA023457 | *ONAC011* |
|  | Degradation of chlorophyll and chloroplast | BGIOSGA003046 | *NYC1* |
|  | Others | BGIOSGA011953 | *OsFBK12* |
| MSTRG.74673.1 | Transcription regulation | BGIOSGA023457 | *ONAC011* |
|  | Degradation of chlorophyll and chloroplast | BGIOSGA003046 | *NYC1* |
|  | Others | BGIOSGA001700 | *salT* |
| MSTRG.5543.1 | Hormone pathways | BGIOSGA019953 | *OsCOI1b* |
|  | Lesion mimic genes | BGIOSGA022234 | *SPL4* |
| MSTRG.11508.1 | Hormone pathways | BGIOSGA013214 | *OsNCED3* |
|  | Degradation of chlorophyll and chloroplast | BGIOSGA010125 | *NOL* |
|  |  | BGIOSGA029383 | *SGRL* |
| MSTRG.20126.1 | Lesion mimic genes | BGIOSGA022234 | *SPL4* |
|  | Others | BGIOSGA011953 | *OsFBK12* |
|  |  | BGIOSGA012281 | *PLS2* |
| MSTRG.26261.1 | Hormone pathways | BGIOSGA019953 | *OsCOI1b* |
|  | Degradation of chlorophyll and chloroplast | BGIOSGA022884 | *NYC3* |
|  |  | BGIOSGA032012 | *OsRCCR1* |
| MSTRG.43455.1 | Transcription regulation | BGIOSGA023457 | *ONAC011* |
|  | Degradation of chlorophyll and chloroplast | BGIOSGA003046 | *NYC1* |
|  |  | BGIOSGA010125 | *NOL* |
|  |  | BGIOSGA029383 | *SGRL* |
| MSTRG.57335.8 | Hormone pathways | BGIOSGA019953 | *OsCOI1b* |
|  |  | BGIOSGA013214 | *OsNCED3* |
|  | Degradation of chlorophyll and chloroplast | BGIOSGA011859 | *OsPAO* |
|  |  | BGIOSGA022884 | *NYC3* |
|  |  | BGIOSGA029383 | *SGRL* |
|  |  | BGIOSGA032012 | *OsRCCR1* |
| MSTRG.7519.1 | Transcription regulation | BGIOSGA023457 | *ONAC011* |
|  | Degradation of chlorophyll and chloroplast | BGIOSGA003046 | *NYC1* |
|  |  | BGIOSGA010125 | *OsNOL* |
| MSTRG.80924.2 | Transcription regulation | BGIOSGA023457 | *ONAC011* |
|  | Degradation of chlorophyll and chloroplast | BGIOSGA003046 | *NYC1* |
|  |  | BGIOSGA010125 | *NOL* |
| MSTRG.53258.3 | Degradation of chlorophyll and chloroplast | BGIOSGA010125 | *NOL* |
|  |  | BGIOSGA029383 | *SGRL* |
